# Effects of Candesartan vs Lisinopril on Neurocognitive Function in Older Adults With Executive Mild Cognitive Impairment: A Randomized Clinical Trial

**DOI:** 10.1001/jamanetworkopen.2020.12252

**Published:** 2020-08-06

**Authors:** Ihab Hajjar, Maureen Okafor, Darius McDaniel, Malik Obideen, Elizabeth Dee, Mahsa Shokouhi, Arshed A. Quyyumi, Allan Levey, Felicia Goldstein

**Affiliations:** 1 Department of Neurology, Emory University School of Medicine, Atlanta, Georgia; 2 Division of General Medicine and Geriatrics, Department of Medicine, Emory University School of Medicine, Atlanta, Georgia; 3 Division of Cardiology, Department of Medicine, Emory University School of Medicine, Atlanta, Georgia

## Abstract

**Importance:**

Observational studies have suggested that angiotensin receptor blockers are associated with a unique cognitive protection. It is unclear if this is due to reduced blood pressure (BP) or angiotensin receptors type 1 blockade.

**Objective:**

To determine neurocognitive effects of candesartan vs lisinopril in older adults with mild cognitive impairment (MCI).

**Design, Setting, and Participants:**

This randomized clinical trial included participants aged 55 years or older with MCI and hypertension. Individuals were withdrawn from prior antihypertensive therapy and randomized in a 1 to 1 ratio to candesartan or lisinopril from June 2014 to December 2018. Participants underwent cognitive assessments at baseline and at 6 and 12 months. Brain magnetic resonance images were obtained at baseline and 12 months. This intent-to-treat study was double-blind and powered for a sample size accounting for 20% dropout. Data were analyzed from May to October 2019.

**Interventions:**

Escalating doses of oral candesartan (up to 32 mg) or lisinopril (up to 40 mg) once daily. Open-label antihypertensive drug treatments were added as needed to achieve BP less than 140/90 mm Hg.

**Main Outcomes and Measures:**

The primary outcome was executive function (measured using the Trail Making Test, Executive Abilities: Measures and Instruments for Neurobehavioral Evaluation and Research tool) and secondary outcomes were episodic memory (measured using the Hopkins Verbal Learning Test-Revised) and microvascular brain injury reflected by magnetic resonance images of white matter lesions.

**Results:**

Among 176 randomized participants (mean [SD] age, 66.0 [7.8] years; 101 [57.4%] women; 113 [64.2%] African American), 87 were assigned to candesartan and 89 were assigned to lisinopril. Among these, 141 participants completed the trial, including 77 in the candesartan group and 64 in the lisinopril group. Although the lisinopril vs candesartan groups achieved similar BP (12-month mean [SD] systolic BP: 130 [17] mm Hg vs 134 [20] mm Hg; *P* = .20; 12-month mean [SD] diastolic BP: 77 [10] mm Hg vs 78 [11] mm Hg; *P* = .52), candesartan was superior to lisinopril on the primary outcome of executive function measured by Trail Making Test Part B (effect size [ES] = −12.8 [95% CI, −22.5 to −3.1]) but not Executive Abilities: Measures and Instruments for Neurobehavioral Evaluation and Research score (ES = −0.03 [95% CI, −0.08 to 0.03]). Candesartan was also superior to lisinopril on the secondary outcome of Hopkins Verbal Learning Test-Revised delayed recall (ES = 0.4 [95% CI, 0.02 to 0.8]) and retention (ES = 5.1 [95% CI, 0.7 to 9.5]).

**Conclusions and Relevance:**

These findings suggest that in older adults with MCI, 1-year treatment with candesartan had superior neurocognitive outcomes compared with lisinopril. These effects are likely independent of the BP-lowering effect of candesartan.

**Trial Registration:**

ClinicalTrials.gov Identifier: NCT01984164

## Key Points

**Question:**

What are the comparative neurocognitive effects of an angiotensin receptor blocker, candesartan, and an angiotensin-converting enzyme inhibitor, lisinopril, on adults with hypertension and executive mild cognitive impairment?

**Findings:**

In this randomized clinical trial of 176 adults with hypertension, 12-month treatment with candesartan was associated with improvement in executive function and episodic memory compared with lisinopril.

**Meaning:**

These findings suggest that among older adults with hypertension and mild cognitive impairment, treatment with candesartan provided better neurocognitive effects compared with lisinopril despite equivalent blood pressure levels.

## Teaser

This randomized clinical trial compares treatment with candesartan vs lisinopril among adults with hypertension and mild cognitive impairment.

## Introduction

Clinical trials have suggested that lowering blood pressure (BP) using antihypertensive therapy provides cognitive protection in individuals without impaired cognition.^1,2^ It is unclear if this is also true in individuals who have symptoms of cognitive impairment. Studies designed to compare the neurocognitive effects of antihypertensive classes in individuals who are cognitively impaired are limited. It is also unclear if these effects are related to the reduction in BP or to possible pleiotropic BP-independent effects of certain antihypertensive medications. Comparing antihypertensive drug classes independent of the degree of BP lowering is critical to determine if these effects on cognition are only secondary to their BP or other non-BP effects. Such pleiotropic effects of antihypertensive drugs may be leveraged to repurpose drugs for cognitive disorders, including Alzheimer disease (AD).

Another limitation of past studies is the use of a global cognitive measure that may not detect significant effects on executive function. Executive function is a key cognitive domain that is highly susceptible to hypertension,^3,4^ and impairment in executive function highly correlates with future functional impairment and dementia.^5^ Executive dysfunction, within the context of hypertension, reflects vascular cognitive impairment and underlying vascular brain injury such as white matter lesions (WMLs) on brain magnetic resonance imaging (MRI).^6^ A 2019 study^7^ suggested the latter may be ameliorated by antihypertensive therapy. However, it is unclear if there are differential effects among antihypertensive classes.

Prior observational studies have suggested that drugs that modulate the renin angiotensin system (RAS) have a cognitive protective association.^8,9,10^ Of the RAS-modulating drugs, angiotensin receptor blockers (ARBs) may have a superior association compared with other classes in affecting cognitive function.^11^ The potential explanation may be related to their differential blockade of the 2 angiotensin receptors (AT_1_ and AT_2_) in the brain.^12,13^ Activation of AT_1_ promotes neurotoxic mechanisms, such as oxidative stress, neuroinflammation, endothelial dysfunction, cerebral hypoperfusion and cholinergic depletion.^14,15,16^ Stimulation of AT_2_ counteracts these mechanisms.^17,18,19^ Therefore, selective blockade of the AT_1_ receptors with ARBs may offer superior protection than simultaneously lowering both of AT_1_ and AT_2_ receptor activities, such as with angiotensin-converting enzyme inhibitors (ACEIs). In our previous pilot study in adults with hypertension and mild cognitive impairment (MCI),^20^ candesartan, an ARB, was superior to lisinopril, an ACEI, in providing cognitive protection.

The objective of this 1-year double-blind randomized clinical trial was to compare neurocognitive effects of candesartan with lisinopril in individuals with MCI and hypertension with the goal of achieving equivalent BP levels in both groups.

## Methods

The trial protocol and informed consent form were reviewed and approved by the Emory institutional review board committee. All study participants provided written informed consent, and participation was in accordance with the principles of the Declaration of Helsinki.^21^ For participants who lacked decision capacity based on a study comprehension quiz, a study surrogate or study partner who could provide consent on the participant’s behalf was required. From the initiation of the trial until the final completed analysis, all authors were blinded to the group assignment. An independent biostatistician conducted open interim analyses and presented the study data to a data and safety monitoring board in closed sessions. This study was conducted from June 2014 to December 2018. This report follows the Consolidated Standards of Reporting Trials (CONSORT) reporting guideline.

### Trial Population

Eligible participants were 55 years or older with MCI of the executive or mixed type and a history of hypertension defined as systolic BP 140 mm Hg or higher, diastolic BP of 90 mm Hg or higher, or receiving antihypertensive medication. Executive MCI was defined by meeting 3 criteria: (1) Montreal Cognitive Assessment score of at least 26^22,23^; (2) a performance in at least the 10th percentile on at least 1 of 5 screening tests of executive function, including the Trail Making Test (TMT) Part B,^24^ modified Stroop Interference Test,^25^ Digit Span Backward and Digit Sequencing,^26^ or Verbal Fluency^27^; and (3) preserved functional ability reflected by a Functional Assessment Questionnaire score of 7 or lower.^28^

Key exclusion criteria were (1) intolerance to any ACEI or ARB, (2) systolic BP higher than 200 mm Hg or diastolic BP higher than 110 mm Hg, (3) elevated baseline serum creatinine level greater than 1.99 mg/dL (to convert to micromoles per liter, multiply by 1) or serum potassium level greater than 5.5 mEq/dL (to convert to micromoles per liter, multiply by 1), (4) any active medical or psychiatric condition requiring medical attention or deemed a safety risk by the study physician (eg, decompensated heart failure, hematologic disease, stroke in the past 3 years [self-reported clinical stroke], or incidental large vessel infarcts on brain MRI), (5) a confirmed clinical diagnosis of dementia (self-report or caregiver report), and (6) inability to perform study procedures. Participants with contraindications or intolerance to MRI were enrolled but did not undergo neuroimaging. Additional eligibility criteria are described in the Trial Protocol in Supplement 1.

### Trial Design

This randomized clinical trial was an investigator-initiated single-center double-blind trial conducted in the metropolitan Atlanta, Georgia, area, comparing the neurocognitive effects of oral candesartan with lisinopril during 1 year. Study participants underwent screening, baseline, 6-month, and 12-month evaluations. Additional visits included biweekly titration visits after randomization to escalate hypertension treatment until goal or maximum drug treatment were achieved. Additional visits with the study physicians were conducted as needed when BP was out of range, adverse events were reported, or at the request of the participant. Visits were supplemented with telephone calls throughout the trial period.

Eligible participants were provided a calibrated BP machine and were trained on using it during the screening visit. All those receiving prestudy antihypertensive medication were guided through a period of gradual taper and washout per a standard protocol. Participants were subsequently randomized in a 1-to-1 block randomization using a computerized random number generator on race (African American vs other) and number of antihypertensive medications prior to study enrollment (≤2 vs >2) to ensure equal distribution between treatment groups and to allow for future preplanned subgroup analyses.

### Interventions

Following baseline and randomization, participants received escalating treatment in identical oral capsule formulations and were evaluated every 2 weeks with the goal of achieving mean sitting BP of less than 140/90 mm Hg. All participants commenced the initial daily dosage of candesartan 8 mg or matched capsule of lisinopril 10 mg. Subsequent dose titration for candesartan was 8 mg to 16 mg to 32 mg and for lisinopril was 10 mg to 20 mg to 40 mg. If BP was still higher than 140/90 mm Hg at the highest dose of the blinded medication, additional open-label antihypertensive therapy was added, starting with hydrochlorothiazide at 12.5 mg to 25 mg, amlodipine 2.5 mg to 5 mg to 10 mg, and metoprolol XL 12.5 mg to 25 mg to 50 mg, until BP control was achieved or all classes were used. Participants received all their antihypertensive therapy from the study site for 1 year from the Emory Investigational Drug Services. Throughout the study, participants did not receive any concomitant BP management from other sources, and all nonstudy medications were reviewed at each visit.

### Trial Procedures

All participants underwent a full history and examination and provided data by interview or direct observation or examination. Collected data included demographic characteristics (including self-identified race/ethnicity), anthropometric information, seated and standing standardized BP measurements (appropriate cuff size, rest period, unclothed arm), and concurrent medication and supplement inventory. A series of comprehensive neuropsychological tests assessing multiple domains was conducted at baseline, 6 months, and 12 months. Executive function was assessed using TMT Parts A and B.^29^ Part A was collected to correct for motor speed and visual-perceptual demands on TMT by subtracting completion time for TMT Part A from completion time for Part B (TMT B − A). Part B − A provides a relatively purer measure of executive functioning.^30^ Participants who could not complete the TMT Part B after 5 minutes were stopped, and their scores were recorded as 300 seconds. A second measure of executive function was collected using the computer-based Executive Abilities: Measures and Instruments for Neurobehavioral Evaluation and Research toolbox.^31,32^ Each participant was asked to complete a set of tasks using a keyboard and computer screen, and data were further analyzed using item response theory to obtain a single composite executive function score.^33^ Episodic memory was assessed using the Hopkins Verbal Learning Test-Revised (HVLT-R),^34^ which included delayed (ie, 20 minutes) recall, retention, and recognition discrimination indices.^34^ Language was assessed using the Boston Naming Test.^35,36^ The Digit Span Test^37^ and Center for Epidemiologic Studies Depression^38^ scale were also collected. Functional status and levels of independence were measured by the Instrumental Activities of Daily Living scale.^39^ The Dysexecutive Functioning Questionnaire was collected for evaluating functional abilities related to executive cognitive function.^40,41^ Physical Activity Scale for the Elderly was used to measure occupational, household, and leisure activities.^42,43^

All MRI scans were performed using a 3T MRI scanner (Magnetom Prisma; Siemens Healthcare) using a 20-channel head coil at baseline and 12 months. The MRI sequences collected included a 3-dimensional fast spoiled gradient recalled echo sequence, a 3-dimensional T2 fluid-attenuated inversion recovery, fast spin echo sequence and arterial spin labeling. The volumetric measures of the hippocampus and other cortical/subcortical regions were extracted using FreeSurfer software version 5.3 (FreeSurfer) from T1MPRAGE structural image. The fluid-attenuated inversion recovery images were used to calculate WML volume using the Lesion Segmentation Tool in Statistical Parametric Mapping.^44^ Details of image acquisition and processing are in the Trial Protocol in Supplement 1.

### Safety

Safety assessments included a physical and neurological examination prior to randomization, periodic assessment of medical history data and clinical measures (eg, sitting and standing BP, heart rate, routine laboratory measurements), and surveillance of adverse events. Significant events identified during the study period through self-report by the participant or next of kin, or clinically significant laboratory results outside of reference ranges were recorded as adverse events. Participants were screened for a new diagnosis of dementia or the use of cholinesterase inhibitors or memantine at each visit.

### Outcomes

The primary outcome for this trial was executive function (ie, TMT and Executive Abilities: Measures and Instruments for Neurobehavioral Evaluation and Research executive composite score) measured 3 times: baseline, 6 months, and 12 months. The secondary outcomes included episodic memory (ie, HVLT-R delayed recall, retention, and recognition discrimination), other cognitive domains (ie, language using the Boston Naming Test and attention using the Digit Span Test) and brain MRI (ie, WML volume, hippocampal volume and global cerebral perfusion).

### Statistical Analysis

All analyses were conducted using the intention-to-treat principle. Baseline characteristics were summarized by randomization group using descriptive statistics including mean (SD) and count (percentage). We performed χ^2^ test on categorical data, and *t* test or Wilcoxon rank test were performed on continuous data. The primary analysis was performed using mixed model with repeated measures. The treatment group was the primary factor of interest. Least-square means were derived from these models. We included the stratification variables (ie, race/ethnicity and number of antihypertensive medications prior to study enrollment) and systolic BP (mean of 2 seated readings as a repeated measure in the model) to assess the drug effects adjusted for systolic BP changes during the study period. Sensitivity analyses were conducted by adjusting for the baseline outcome measures for those that differed at baseline between the 2 groups. Results are provided as adjusted least-square means with SE and effect size (ES) estimates (calculated from mixed model with repeated measures as the adjusted difference in cognitive change between the 2 groups over the study period). Time was included as a continuous variable (with T1 indicating baseline; T2, 6 months; and T3, 12 months) with 2-sided 95% CIs as recommended by the updated CONSORT guidelines for continuous outcomes.^45^ Additional details are provided in the Trial Protocol in Supplement 1.

Using a 2-sided α = .05, an estimated sample size of 140 would provide 80% power to detect at least an ES of 0.24 on executive function (TMT Part B − A) in our pilot study.^20,46^ To account for a dropout rate of 20%, we preplanned to enroll approximately 175 participants. Interim analysis with the data and safety monitoring board was completed by an independent statistical team when 50% of the trial sample finished 12-month visits. We used an α spending function approach with O’Brien-Fleming sequential boundaries (α levels at .0056 and .0482) to preserve the overall 2-sided α error rate of .05.^47^ The data and safety monitoring board recommended continuation and completion of the study after the interim analysis. Statistical analyses were performed with SAS statistical software version 9.4 (SAS Institute). Data were analyzed from May to October 2019.

## Results

**Figure 1.**
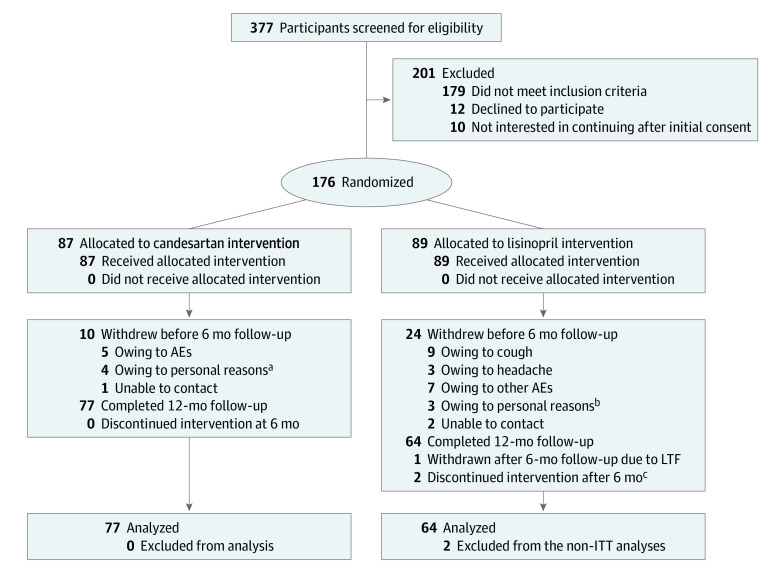
Flow Diagram of Study Recruitment AE indicates adverse event; ITT, intention-to-treat; and LTF, loss to follow-up. ^a^Personal reasons for withdrawing from candesartan group included: 1 owing to depression, 2 owing to insufficient study compensation to continue, and 1 owing to high home blood pressure readings. ^b^Personal reasons for withdrawing from lisinopril group included 1 owing to relocation, 1 owing to busy life schedule, and 1 owing to insufficient study compensation. ^c^Two participants discontinued study intervention at 7 months owing to cough and owing to hospitalization for lung tumor resection. The latter was receiving lisinopril as part of their clinical treatment at 12 months. Both participants completed 12-month follow-up visit.

Of 377 individuals screened, 176 (47%) were eligible for the study, agreed to proceed with tapering of previously prescribed antihypertensive therapy, and were randomized to candesartan or lisinopril. Of those randomized, 33 dropped out before the 6-month assessment, and an additional 2 participants dropped out after the 6-month assessment. Two participants completed the study but had their intervention discontinued after 6 months owing to hospitalization for resection of a lung mass or a persistent cough. The final sample for the intention-to-treat analysis was 141 individuals, including 77 in the candesartan group and 64 in the lisinopril group. Figure 1 shows the CONSORT flowchart for this trial.

### Baseline Characteristics

Among 176 randomized participants, including 87 assigned to candesartan and 89 assigned to lisinopril, the mean (SD) age was 66.0 (7.8) years 101 (57.4%) were women, and 113 were African American (64.2%). Baseline group comparisons are shown in the Table. There was no difference between the 2 groups in the qualifying task for executive dysfunction during the screening process. Participants assigned to either lisinopril or candesartan were also similar on all baseline characteristics, including cognitive performance, except for the baseline TMT Part B (mean [SD] score, 130.6 [78.7] seconds vs 156.0 [8.0] seconds) and Part B − A (mean [SD] score, 92.3 [74.0] seconds vs 115.4 [79.4] seconds). When we divided the group into those who scored 300 seconds on the TMT Part B at baseline (the maximum allowable time to finish the task), there was no difference in the number of participants who scored 300 seconds between the lisinopril group vs candesartan group (12 individuals [13.5%] vs 18 individuals [20.7%]). The mean (SD) TMT Part B score in those with a score less than 300 seconds was 104.2 (43.9) seconds in the lisinopril group vs 118.4 (49.4) seconds in the candesartan group, and the mean (SD) score for TMT Part B − A was 67.3 (58.2) seconds in the lisinopril group vs 80.8 (44.7) seconds in the candesartan group (*P* = .06). The TMT analyses were adjusted for baseline performances.

**Table.** Baseline Clinical, Demographic, Cognitive, and Neuroimaging Characteristics Comparisons Between Lisinopril and Candesartan Treatment Groups

Characteristic	No. (%)	
	Lisinopril (n = 89)	Candesartan (n = 87)
Age, mean (SD), y	65.8 (7.2)	66.1 (8.3)
Women	54 (60.7)	47 (54.0)
Ethnicity		
Not Hispanic or Latino	83 (94.3)	84 (96.6)
Hispanic or Latino	5 (5.7)	3 (3.4)
Race		
White	31 (34.8)	29 (33.3)
African American	57 (64.0)	56 (64.4)
Other	1 (1.1)	2 (2.3)
Education, mean (SD), y	15.4 (2.5)	14.7 (2.6)
Marital status		
Married	33 (37.9)	30 (35.3)
Divorced or separated	32 (36.8)	32 (37.6)
Widowed	14 (16.1)	12 (14.1)
Single or never married	7 (8.0)	10 (11.8)
Other	1 (1.1)	1 (1.2)
Residence situation		
Alone	33 (37.1)	34 (39.1)
With spouse or partner	35 (39.3)	36 (41.4)
With child or other family	16 (18.0)	15 (17.2)
Other	5 (5.6)	2 (2.3)
BMI, mean (SD)	32.6 (7.3)	32.5 (6.9)
Family history of dementia	69 (79.3)	56 (67.5)
BP, mean (SD), mm Hg		
Systolic BP		
Sitting	145.3 (21.0)	140.2 (21.1)
Standing, at 3 min	150.9 (22.4)	146.7 (22.2)
Diastolic BP		
Sitting	85.2 (13.2)	83.7 (12.9)
Standing, at 3 min	92.0 (14.0)	90.9 (14.2)
Pulse, mean (SD), beats per min		
Sitting	70.7 (11.8)	69.6 (10.7)
Standing, at 3 min	78.6 (13.5)	78.1 (12.6)
Cognitive status, mean (SD)		
MoCA score	21.7 (3.5)	21.4 (3.4)
TMT		
Part A, completion time, s	38.2 (14.7)	40.6 (15.8)
Part B, completion time, s	130.6 (78.7)	156.0 (86.0)
Part B − A, s	92.3 (74.0)	115.4 (79.4)
HVLT-R score		
Immediate recall	22.8 (4.4)	21.5 (4.7)
Delayed recall	7.0 (3.2)	6.5 (3.1)
Recognition discrimination index	11.2 (1.1)	10.8 (1.9)
Retention	76.0 (30.4)	71.1 (30.7)
EXAMINER executive composite score	0.1 (0.5)	−0.0 (0.6)
Boston Naming Test score	13.7 (1.4)	13.1 (2.0)
Digital span test score		
Forward	8.9 (2.0)	8.9 (2.1)
Backward	5.2 (1.9)	5.0 (1.9)
CES-D score	10.1 (8.7)	11.2 (9.6)
PASE score	193.3 (162.7)	211.3 (194.1)
Dysexecutive Questionnaire score	12.2 (9.2)	14.3 (11.5)
Instrumental Activities of Daily Living score	7.7 (1.0)	7.9 (2.0)
Functional Activities Questionnaire score	0.7 (1.0)	0.9 (1.6)
MCI category		
Executive	56 (62.9)	43 (49.4)
Mixed, executive and amnestic	33 (37.1)	44 (50.6)
Executive dysfunction-qualifying task during screening		
TMT part B	35 (39.3)	41 (47.1)
Verbal fluency	28 (31.5)	27 (31.0)
Stroop interference test	33 (37.1)	41 (47.1)
Digit span (backward)	64 (71.9)	58 (66.7)
Digit sequencing	18 (20.2)	13 (14.9)
Blood chemistry levels, mean (SD)		
Hemoglobin, g/dL	13.3 (1.4)	13.4 (1.2)
Sodium, mEq/L	140.3 (2.3)	140.1 (2.7)
Potassium, mEq/L	4.1 (0.6)	4.1 (0.5)
Creatinine, mg/dL	0.9 (0.3)	0.9 (0.2)
Comorbidities		
Diabetes	27 (30.7)	24 (27.6)
Heart disease (coronary or valvular)	29 (32.6)	18 (20.7)
Hyperlipidemia	46 (52.9)	54 (62.8)
Remote stroke^a^	6 (6.7)	5 (5.7)
Depression^b^	23 (26.1)	22 (25.3)
Prerandomization medications		
ACEI	32 (36.0)	33 (37.9)
ARB	20 (22.5)	17 (19.5)
Either ACEI or ARB	51 (57.3)	50 (57.5)
Any other antihypertensive medication	72 (80.9)	79 (90.8)
Cholinesterase inhibitors or memantine	3 (3.4)	1 (1.1)
Antidiabetes	24 (27.0)	23 (26.4)
Lipid-lowering agent	36 (40.4)	48 (55.2)
Antidepressant	21 (23.6)	19 (21.8)
Neuroimaging measures		
No.	75	74
Whole brain cerebral blood flow, mean (SD), mL/100 g/min	48.0 (7.9)	46.9 (8.7)
WML volume, mean (SD), mm^3^	4.8 (8.4)	2.3 (2.3)
Hippocampal volume, mean (SD), mm^3^	7213 (917)	7375 (1084)

Abbreviations: ACEI, angiotensin-converting enzyme inhibitors; ARB, angiotensin II receptor blockers; BMI, body mass index (calculated as weight in kilograms divided by height in meters squared); BP, blood pressure; CES-D, Center for Epidemiologic Studies Depression scale; EXAMINER, Executive Abilities: Measures and Instruments for Neurobehavioral Evaluation and Research; HVLT-R, Hopkins Verbal Learning Test-Revised; MCI, mild cognitive impairment; MoCA, Montreal Cognitive Assessment; PASE, Physical Activity Scale for the Elderly; TMT, Trail Making Test.

SI conversion factors: To convert creatinine to micromoles per liter, multiply by 76.25; hemoglobin to grams per liter, multiply by 10; and potassium and sodium to millimoles per liter, multiply by 1.

^a^ Remote stroke occurring more than 3 years from study enrollment.

^b^ Clinically diagnosed depression.

Among individuals randomized to candesartan, 44 individuals (50.6%) had mixed executive and amnestic-type MCI vs 33 individuals (37.1%) in the lisinopril group (*P* = .07). There was no difference in pre-enrollment use of ARBs or ACEIs by randomization group. There was also no difference between those who completed the study and those who dropped out before the 12-month assessment (eTable 1 in Supplement 2). There was no difference in the number of participants who completed vs dropped out of the candesartan or lisinopril treatment groups.

### Intervention Descriptions and BP

**Figure 2.**
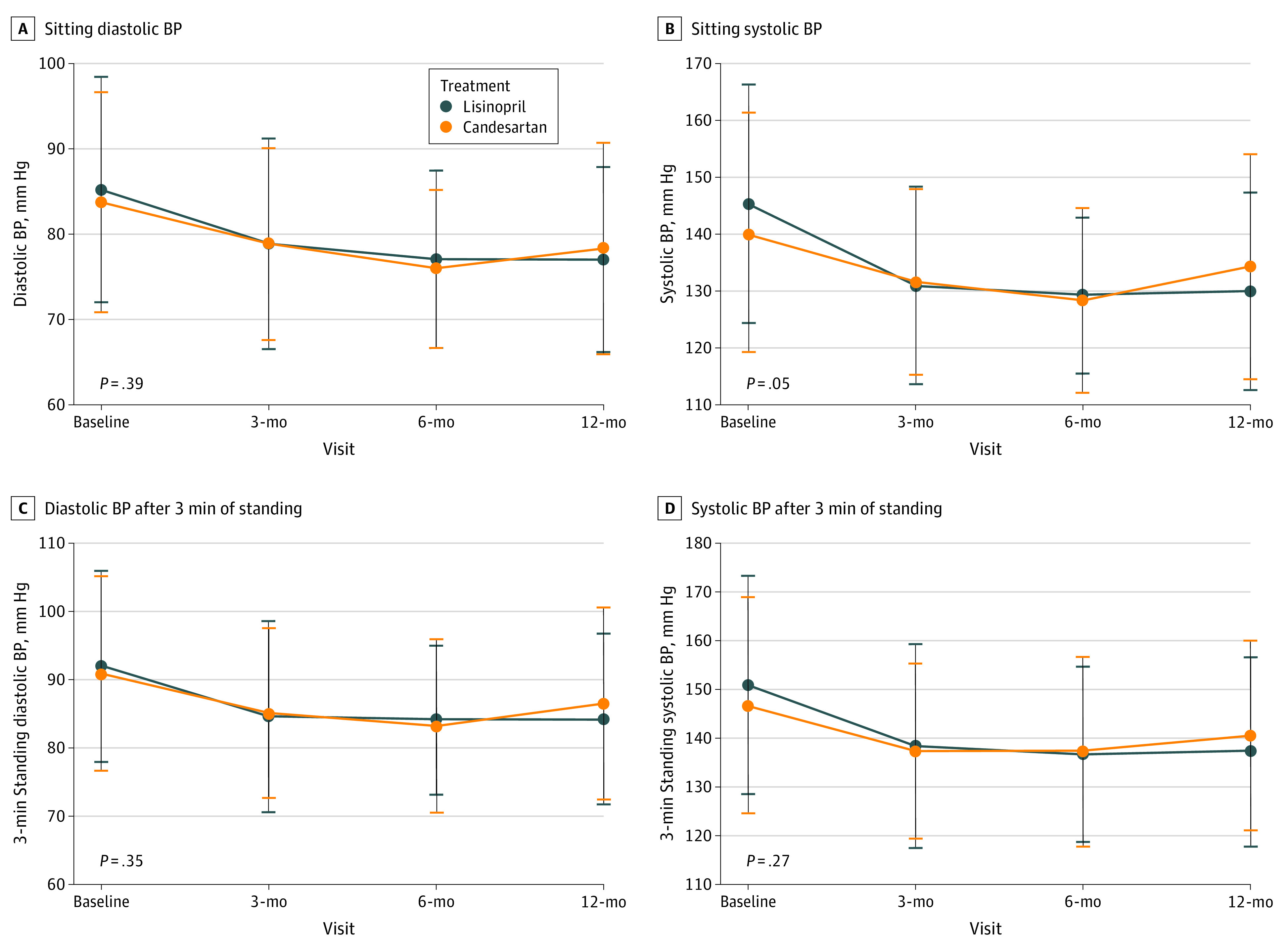
Mean Sitting and Standing Systolic and Diastolic Blood Pressure (BP) Over Study Period, by Treatment Group *P* values are obtained from mixed model repeated measure for the interaction of treatment effect over time. Whiskers indicate SDs.

Nearly half of the study sample (86 participants [48.9%]) achieved BP control on the initial dose of candesartan (8 mg) or lisinopril (10 mg), and 46 participants (26.1%) needed an additional agent after maximal titration of study medications. The distribution of candesartan or lisinopril doses and of the additional open-label antihypertensive medication doses were similar between the 2 groups (eTable 2 in Supplement 2). Both groups achieved equivalent BPs during the study with no difference in either sitting or standing systolic and diastolic BP at baseline (ie, without any treatment) or during the follow-up period. At the 12-month visit, mean (SD) systolic BPs were 130 (17) mm Hg for lisinopril vs 134 (11) mm Hg for candesartan (*P* = .20), and mean (SD) diastolic BPs were 77 (10) mm Hg for lisinopril vs 78 (11) mm Hg for candesartan (*P* = .52). Similarly, serum potassium and creatinine levels did not differ between the 2 groups. These results are shown in Figure 2 and eTable 3 and the eFigure in Supplement 2.

### Adverse Events

More participants reported cough in the lisinopril group compared with the candesartan group (24 participants [27.0%] vs 7 participants [8.0%]; *P* = .005), but since both groups experienced cough, this did not lead to unblinding of the study team. The candesartan group reported more skin rash than the lisinopril group (8 participants [9.1%] vs 1 participant [1.1%]; *P* = .008). There were no differences in other adverse events reported during the study (eTable 4 in Supplement 2). More participants in the lisinopril group withdrew from the study owing to adverse events compared with the candesartan group (25 participants [28.1%] vs 10 participants [11.5%]; *P* = .01) (Figure 1).

### Cognitive Outcomes

**Figure 3.**
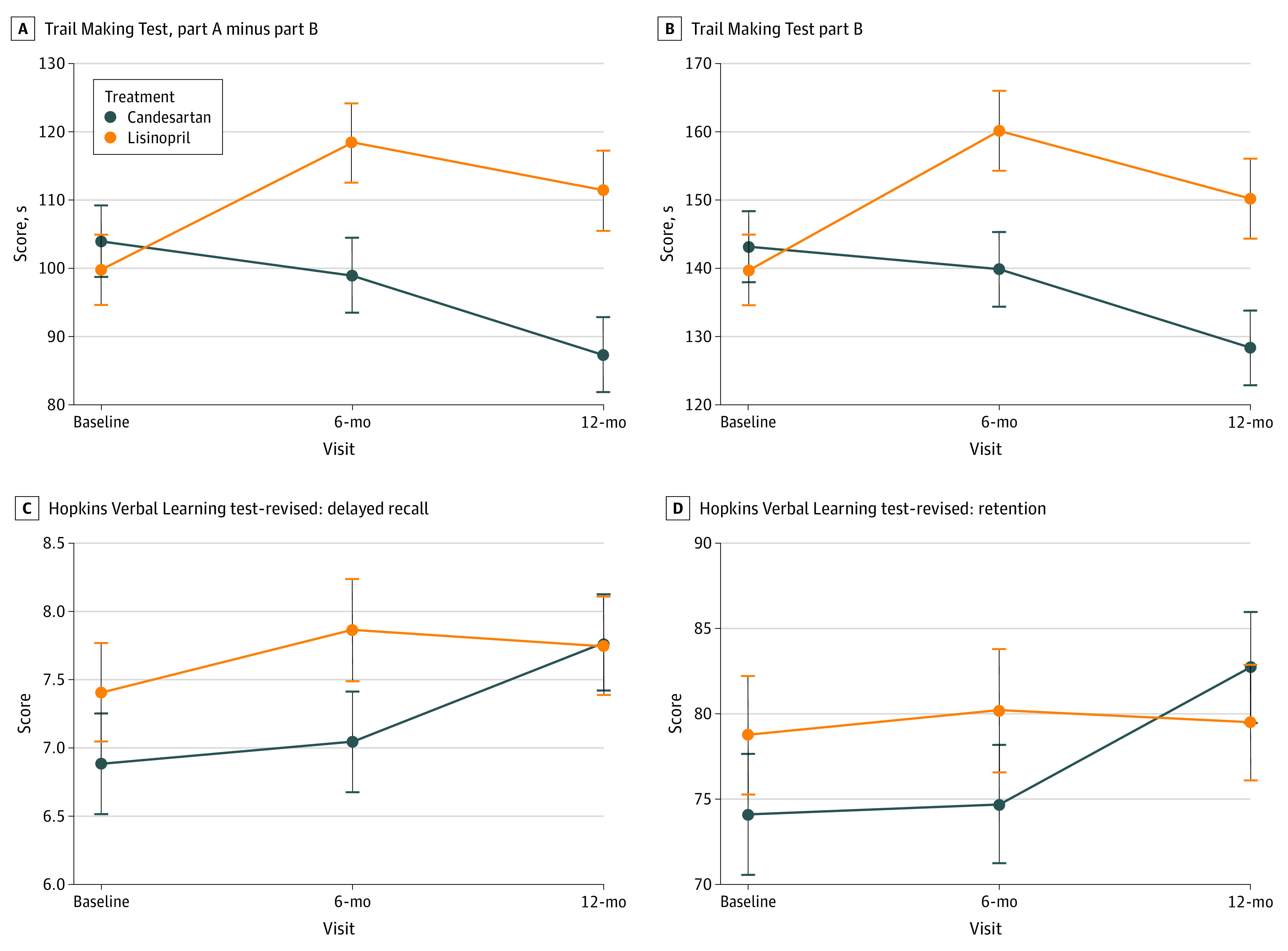
Change in Trail Making Test Parts B and B − A, and Hopkins Verbal Learning Test-Revised, Delayed Recall and Retention During 12 Months Stratified by Treatment Group Values are model-derived least-square means and SEs (whiskers) from mixed model repeated measure and are adjusted for systolic blood pressure and stratification variables. Trail Making Test analyses are also adjusted for baseline Trail Making Test scores. *P* values for the treatment effect are obtained from the mixed model repeated measure models comparing change over the study period (visit as a continuous measure: 1 = baseline, 2 = 6 months, and 3 = 12 months) between the 2 groups: A, *P* = .004; B, *P* = .01; C, *P* = .04; D, *P* = .02.

After adjusting for systolic BP and stratification variables, participants randomized to candesartan demonstrated a significant improvement in executive function compared with those randomized to lisinopril, who showed decline, measured by TMT Part B (ES = −12.0 [95% CI, −21.7 to −2.3]) seconds; *P* = .01) and TMT Part B − A (ES = −13.6 [95% CI, −23.6 to −3.7] seconds; *P* = .008). Since TMT Part B was different between groups at baseline, further analysis was conducted to adjust for baseline performance on this task. The baseline-adjusted analyses showed similar results (TMT Part B: ES = −12.8 [95% CI, −22.6 to −3.1] seconds; *P* = .01; TMT Part B − A: ES = −14.3 [95% CI, −24.2 to −4.5]; *P* = .004). At the 12-month visit, the adjusted TMT Part B and B − A were significantly better in the candesartan group compared with the lisinopril group (TMT Part B: mean [SE] score, 128.3 [5.4] seconds vs 150.2 [5.9] seconds; difference, 22.3 [95% CI, 7.3 to 37.3] seconds; *P* = .004; TMT Part B − A: mean [SE] score, 87.2 [5.5] seconds vs 111.4 [5.9] seconds; difference, 24.6 [95% CI, 9.5 to 39.7] seconds). There were no differences between treatment groups in Executive Abilities: Measures and Instruments for Neurobehavioral Evaluation and Research scores, our second executive function measure (ES = −0.03 [95% CI, −0.08 to 0.03] seconds; *P* = .31) (Figure 3A and B; eTable 5 in Supplement 2).

Treatment with candesartan had a superior effect on episodic memory, measured by the delayed recall and retention indices of the HVLT-R compared with lisinopril after adjusting for systolic BP and stratification variables. The ES for delayed recall was 0.4 (95% CI, 0.02 to 0.8; *P* = .04), and the ES for retention was 5.1 (95% CI, 0.7 to 9.5; *P* = .02) (Figure 3C and D); eTable 5 in Supplement 2). We did not observe group differences in the additional secondary cognitive outcomes of the Boston Naming Test and Digit Span Tests or in the functional measures of Instrumental Activities of Daily Living or Dysexecutive Functioning Questionnaire (eTable 5 in Supplement 2).

### MRI Outcomes

Of 176 randomized participants, 27 did not complete an MRI scan at baseline (15 had metal implants with either pacemaker, stent, or inferior vena cava filter and 12 were claustrophobic). Of 141 participants who returned for their 12-month visit, an additional 8 participants refused to have a follow-up MRI. The final sample with both baseline and 12-month MRI was 104 participants (73.8%), including 51 participants in the candesartan group and 53 participants in the lisinopril group.

**Figure 4.**
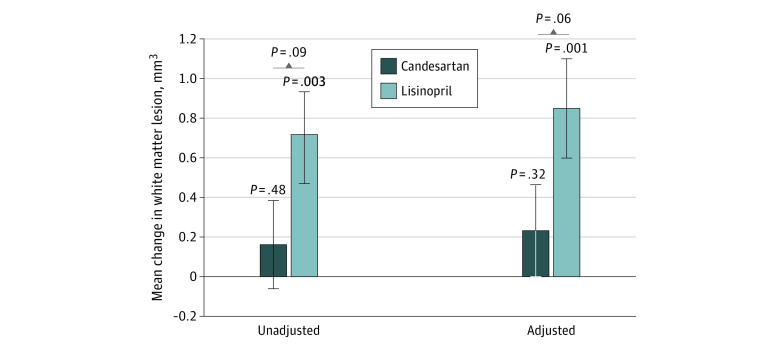
Change in White Matter Lesion Volumes From Baseline to 12 Months Stratified by Treatment Group Adjusted values are least-square means and SEs (whiskers) obtained from mixed model repeated measure and are adjusted for systolic blood pressure, participant race/ethnicity, and prestudy antihypertensive medications.

At baseline, the lisinopril group had a mean (SD) WML volume of 4.8 (8.4) mm^3^, and the candesartan group had a mean (SD) of 2.3 (2.3) mm^3^. After adjusting for BP and stratification variables, those randomized to candesartan had less WML accumulation of 0.2 (95% CI, −0.2 to 0.7) mm^3^ vs 0.8 (95% CI, 0.3 to 1.4) mm^3^ in the lisinopril group (ES = −0.3 [95% CI, −0.6 to 0]). Correcting for baseline levels did not alter the results significantly (change in candesartan group: 0.1 [95% CI, −0.3 to 0.5] mm^3^; change in the lisinopril group: 0.6 [95% CI, 0.2 to 1.0] mm^3^; ES = −0.2 [95% CI, −0.5 to 0]). The results of WML change are shown in Figure 4. There was no significant difference in hippocampal volume (although both groups showed a significant loss of hippocampal volume) or cerebral perfusion by treatment group (eTable 5 in Supplement 2).

## Discussion

In this randomized clinical trial of individuals with MCI and hypertension, 1-year therapy with candesartan was associated with better cognitive outcomes compared with lisinopril, including 1 of the 2 executive function measures, and in memory. Those randomized to lisinopril had significant accumulation of WML, whereas those randomized to candesartan had no significant increase in WML. These effects were independent of BP-lowering properties.

In the largest observational study to our knowledge, use of ARBs was associated with lower risk of dementia and Alzheimer disease (AD) compared with ACEIs and other antihypertensives.^10^ Analysis of the Alzheimer Disease Neuroimaging Initiative data^48^ suggests that people who use ARBs have better cognitive trajectories over 3 years and less WML accumulation. More recently, Ding and colleagues,^49^ in a participant-level meta-analysis of cohort observational studies of predominantly White individuals, reported no differences in antihypertensive classes and the risk of dementia or AD. Although no specific comparisons between ACEI and ARB use was presented, that study did not seem to account for BP change over a long period. The hypertension group excluded those on treatment with controlled BP and did not investigate the association with executive function. This study, in contrast, suggests that when the BP effects are matched in a clinical trial, ARBs provide neurocognitive protection on executive function even over the short-term. This is the second clinical trial in hypertension and MCI suggesting these favorable effects.^46^ However, in this trial we also observed that candesartan may positively affect memory, which suggests a role in amnestic disorders, such as AD.

Prior trials have suggested that lowering of BP is protective against cognitive decline. Only a few of these were designed to compare drug regimens and most excluded individuals with symptomatic cognitive impairment. Two large trials comparing ACEI and ARB included cognitive outcomes: Ongoing Telmisartan Alone and in Combination With Ramipril Global Endpoint Trial, which compared ACEI, ARB, and the combination, and Telmisartan Randomized Assessment Study in ACE Intolerant Subjects With Cardiovascular Disease trial, which compared a placebo with ARB in participants who were intolerant to ACEIs.^50^ Neither of these studies included cognitively symptomatic individuals, and the cognitive measure was a single test of global cognition, the Mini-Mental State Exam, which is rather insensitive to executive impairment and in detecting change over time. Hence, our study addresses a key gap in the literature. Although further larger and longer-term studies are needed, this randomized clinical trial provides additional and incremental support to the positive neurocognitive effects of candesartan that are likely independent and additive to their BP-lowering effects.

Based on our current understanding of the brain RAS activity, we propose that the neurocognitive benefits of candesartan are due to the selectivity of the blockade of the AT_1_ receptor, allowing the AT_2_ receptor to remain active. Supporting evidence for AT_2_ receptor activation comes from animal models in which AT_2_ receptor agonists decreased brain infarct area after ischemic injury by increasing cerebral perfusion in the penumbra,^17^ decreased superoxide production,^17^ and decreased inflammation and axonal degeneration.^19,51^ Activation of the AT_2_ receptor is associated with axonal regeneration, neuronal repair,^52^ and decreases in vascular inflammation.^19^ Additionally, ARBs reduce inflammatory markers more than ACEIs^53^ and potentially restore endothelial dysfunction via AT_2_ receptor stimulation.^54,55,56^ These potential mechanisms should be the focus of future investigations and analyses of this trial data.

Executive function is an important domain that is highly susceptible to vascular brain injury. Hypertension and executive impairment are associated with microvascular brain damage, and our study provides initial evidence that candesartan, but not lisinopril, protects against WML accumulation and vascular brain injury. However, it is worth noting that the between group difference was not statistically significant.

The advantages of this study are the inclusion of participants with MCI and comparing 2 drugs that modulate RAS differently, hence providing important insights into the role of RAS in the brain in individuals who are cognitively impaired. Evaluating measures of executive function, the domain most likely to be affected by hypertension and microvascular disease, provides additional unique insights into the hypertension-cognition-RAS axis. In addition, we recruited a highly diverse population and achieved similar BP levels in both treatment groups.

### Limitations

A limitation of this study is its 1-year study period. Although we have found a positive effect on individual cognitive domains, translation into dementia care should be interpreted within that short-term context. Although executive function has been identified as risk factor for future functional decline, our study did not demonstrate an effect on our functional measures (ie, Dysexecutive Functioning Questionnaire or Instrumental Activities of Daily Living).^57^ This is likely owing to the duration of the study combined with a possible ceiling effect on these measures, since our sample was functionally independent at baseline. Longer study duration may offer greater insight into the functional effects of candesartan. Further, this study cannot be generalizable to other drugs within the same classes of ARB and ACEI, and our findings are applicable to a single drug effect within a class, and not a class effect. Additional limitations include the relatively small sample size, which limited our power to correct for multiple comparisons. Approximately 25% of the clinical trial sample did not have neuroimaging data. Although we were able to detect a possible difference in microvascular disease, our ability to examine the effect on hippocampal volume or cerebral perfusion is likely limited by the power of the neuroimaging component and the 1-year duration of the study. Although we have provided analytical approaches to account for the observed differences at baseline in TMT Part B and WML, our results should be interpreted within the context of this limitation. Nevertheless, our study provides additional evidence for the potential role of ARBs in neurocognitive protection. While these findings would fall into hypotheses generation and justify larger trials, ARBs in general, and candesartan in particular, offer an intriguing therapeutic possibility for cognitive disorders in relation to vascular brain injury, and especially when considered cumulatively with prior observational studies^10^ and our prior pilot.^20^ Currently, there are no available drugs for executive MCI or other cognitive disorders that are becoming more prevalent as populations age. Hence, further investigations of ARBs in this area are critical.

## Conclusions

The results of this randomized clinical trial demonstrate that candesartan is superior to lisinopril on executive function and memory outcomes in MCI. These effects are independent of but likely additive to their BP-lowering effects. Future studies in executive and memory impairments, such as those with prodromal AD, are warranted.

## Notes

Supplement 1. **Trial Protocol**

Supplement 2. **eTable 1.** Clinical, Demographic, and Cognitive Characteristics Comparisons Between Participants Who Completed the Study vs Those Who Dropped Out **eTable 2.** Participants Using Study and Open-Label Antihypertensive Medications **eTable 3.** Blood Pressure, Pulse Rate, and Blood Biochemistry During the Follow-Up Period **eTable 4.** Adverse Events Reported During Trial Period by Treatment Group **eTable 5.** Primary and Secondary Outcomes and Treatment Effects From the Final Mixed Model With Repeated Measures Models **eFigure.** Serum Potassium and Creatinine Levels Stratified by Treatment Group

Supplement 3. **Data Sharing Statement**
