# Supplementary material for: Effects of Candesartan vs Lisinopril on Neurocognitive Function in Older Adults With Executive Mild Cognitive Impairment: A Randomized Clinical Trial
Source: JAMA Netw Open. 2020 Aug 6;3(8):e2012252. doi: 10.1001/jamanetworkopen.2020.12252 (PMC7411539; doi:10.1001/jamanetworkopen.2020.12252)
Supplement: Supplement 1. — Trial Protocol [file jamanetwopen-3-e2012252-s001.pdf]

CAndesartan vs LIsinopril effects on the BRain and Endothelial function in eXecutive MCI (CALIBREX)

(ClinicalTrials.gov Identifier: NCT01984164)

This supplement contains the following items:

1. Final protocol (version 17.0; January 7, 2020)
2. Statistical analysis plan

PROTOCOL TITLE

CAndesartan vs LIsinopril effects on the BRain and Endothelial function in eXecutive MCI (CALIBREX)

VERSION 17, 1/7/2020

Principal Investigator:

Ihab Hajjar, MD
Emory University

Supported by:

The National Institute on Aging

R01AG042127

TABLE OF CONTENTS

STUDY TEAM ROSTER **4**

PARTICIPATING STUDY SITES **6**

PRÉCIS **7**

1. STUDY OBJECTIVES **8**

2. BACKGROUND AND RATIONALE **8**

2.1 Background........ 8

2.2 Study Rationale........ 10

2.3 Prior Studies........ 10

3. STUDY DESIGN **10**

4. SELECTION AND ENROLLMENT OF PARTICIPANTS **10**

4.1 Inclusion Criteria 10

4.2 Exclusion Criteria 11

4.3 Study Recruitment and Payment 11

5. STUDY INTERVENTIONS **12**

5.1 Interventions, Administration, and Duration 12

5.2 Handling of Study Interventions........ 12

5.3 Concomitant Interventions 12

5.4 Allowed Interventions 12

5.5 Required Interventions........ 12

5.6 Prohibited Interventions 12

5.7 Adherence Assessment 12

6. EXPERIMENTAL PROCEDURES AND MEASURES **13**

6.1 Questionnaires and Lifestyle and Anthropmetric Measures........ 13

6.2 Blood Pressure Measurement 13

6.3 Neuropsychological Assessment 13

6.4 Brain MRI Imaging Protocol........ 14

6.5 Vascular Measures........ 16

6.6 Blood Chemistries, Blood Count, and APOE........ 17

7. STUDY SCHEDULE **17**

7.1 Schedule of Evaluations 17

7.2 Description of Evaluations. 17

8. SAFETY ASSESSMENTS **18**

8.1 Potential Risks........ 19

8.2 Specification of Safety Parameters........19
8.3 Antihypertensive Replacement Protocol........20
8.4 Methods and Timing for Assessing, Recording, and Analyzing Safety Parameters........21
8.5 Adverse Events and Serious Adverse Events identification and reporting........21
8.6 Incidental Findings........24
8.7 Follow-up for Adverse Events........24
8.8 Safety Monitoring........24

9. INTERVENTION DISCONTINUATION........24

10. STATISTICAL CONSIDERATIONS........25

10.1 General Design........25
10.2 Sample Size and Randomization........25
10.3 Interim Analyses........25
10.4 Outcomes........26
10.5 Data Analyses........26
10.6 Sample Size Estimates........28

11. DATA COLLECTION AND QUALITY ASSURANCE........28

11.1 Data Collection Forms........28
11.2 Data Management........28
11.3 Quality Assurance........28

12. PARTICIPANT RIGHTS AND CONFIDENTIALITY........29

12.1 Institutional Review Board (IRB) Review........29
12.2 Informed Consent Forms........29
12.3 Cognitive Impairment, Capacity Assessment, and Study Informants and Surrogates........29
12.4 Participant Confidentiality........29
12.5 Study Discontinuation........30

13. PUBLICATION OF RESEARCH FINDINGS........30

14. COMMITTEES........30

15. REFERENCES........31

STUDY TEAM ROSTER

Ihab Hajjar, MD, MS (Principal Investigator)
Section of Geriatrics and Gerontology
Department of Medicine
Emory University
ihajjar@emory.com

Allan Levey, MD, PhD (Co-investigator, Neurologist)
Department of Neurology
Emory University
ALEVEY@emory.edu

Arshed Ali Quyyumi, MD (Co-investigator, Cardiology)
Division of Cardiology
Department of Medicine
Emory University
aquyyum@emory.edu

Felicia Goldstein, PhD (Co-investigator, Neuropsychologist)
Department of Neurology
Emory University
fgoldst@emory.edu

John Hanfelt, PhD (Co-investigator; Statistician)
Department of Biostatistics and Bioinformatics, Rollins School of Public Health, Emory University,
Atlanta, GA Atlanta, GA 30322-4201
jhanfel@emory.edu

Joel Kramer, PsyD (Consultant, Neuropsychologist)
Memory and Aging Center
University of California San Francisco
Jkramer@memory.ucsf.edu

PARTICIPATING STUDY SITES

Wesley Woods Health Center

Emory University Hospital Clinical Research Network

PRÉCIS

Study Title
Candesartan vs Lisinopril for executive mild cognitive impairment

Acronym
CALIBREX

Objectives
Aim 1: Investigate the effect of candesartan compared to lisinopril on the progression of cognitive function evaluated by a battery of neuropsychological tests assessing executive function, memory, attention and cognitive speed in hypertensive older adults with executive mild cognitive impairment.

Aim 2: Investigate the effect of candesartan compared to lisinopril on cerebral perfusion, cerebrovascular reserve (vasoreactivity to carbon dioxide) assessed by arterial spin labeling MRI and microvascular brain injury reflected by diffusion tensor imaging measures of connectivity.

Aim 3: Identify underlying mechanisms explaining the effects of candesartan on neuroimaging (AIM 3A) and cognitive (AIM 3B) outcomes relative to lisinopril including effects on arterial stiffness, atherosclerosis (carotid intima- media thickness, CIMT), and vascular inflammatory and endothelial markers.

Design and Outcomes
Our overall aim is to conduct a 1-year double blind randomized control trial comparing candesartan to lisinopril in individuals with hypertension and executive mild cognitive impairment. Our measures include cognitive function, cerebral perfusion and reserve, markers of vascular brain damage, atherosclerosis, arterial stiffness, vascular inflammation and endothelial function.

Interventions and Duration
The intervention includes candesartan or lisinopril with add-on antihypertensive medications to achieve blood pressure control to below 140/90 mm Hg. The duration of the study is 1 year.

Sample Size and Population
Our target sample size is 140. To achieve this sample size, **500** individuals will be screened to account for screen fails and drop-outs. Individuals will be recruited from the greater Atlanta area to achieve a final sample of 140 (70 in candesartan group, 70 in lisinopril group). Our target population is subjects: 55 years or older, blood pressure (BP) >140/90 mm Hg or receiving antihypertensive medications, with executive MCI.

1. STUDY OBJECTIVES

Our overall aim is to conduct a 1-year double blind randomized control trial comparing candesartan to lisinopril in 140 individuals with hypertension and executive mild cognitive impairment. Our specific aims are:

Aim 1: Investigate the effect of candesartan compared to lisinopril on the progression of cognitive function evaluated by a battery of neuropsychological tests assessing executive function, memory, attention and cognitive speed in hypertensive older adults with executive mild cognitive impairment.

Hypothesis 1: Candesartan is associated with less decline in executive function compared to lisinopril.

Aim 2: Investigate the effect of candesartan compared to lisinopril on cerebral perfusion, cerebrovascular reserve (vasoreactivity to carbon dioxide) assessed by arterial spin labeling MRI and microvascular brain injury reflected by diffusion tensor imaging measures of connectivity.

Hypothesis 2: Candesartan is associated with less decline in cerebral perfusion, vasoreactivity to carbon dioxide, and evidence of microvascular brain injury (white matter integrity) compared to lisinopril.

Aim 3: Identify underlying mechanisms explaining the effects of candesartan on neuroimaging (AIM 3A) and cognitive (AIM 3B) outcomes relative to lisinopril including effects on arterial stiffness, atherosclerosis (carotid intima- media thickness, CIMT), and vascular inflammatory and endothelial markers.

Hypotheses 3: Relative to lisinopril, treatment with candesartan is associated with lower CIMT, arterial stiffness, and inflammatory and endothelial function markers at 12 months, which in turn are associated with improved cerebral perfusion, vasoreactivity, and white matter integrity measures (Hypotheses 3A) as well as less decline in executive function (Hypotheses 3B) at 12 months.

2. BACKGROUND AND RATIONALE

2.1 Background

1- *Hypertension and executive function*: It is estimated that nearly 30% of older adults and 50% of older hypertensives have executive dysfunction, which is generally undetected.[1-3] Executive function refers to cognitive operations that control and coordinate planning and performing complex tasks.[4] It can be viewed as the composite of subdomains including set shifting, working memory, fluency, and processing speed.[4] It reflects the functioning of a broad network of neural structures, with particular involvement of the dorsolateral prefrontal cortex.[5] This brain region and its white matter connections are highly susceptible to vascular injury.[6] Executive dysfunction without dementia belongs to a larger construct of cognitive disorders in the context of vascular brain injury.[7-12] Multiple terms have been suggested for this pattern including non-amnesic mild cognitive impairment (MCI), executive MCI or mild vascular cognitive impairment (VCI).[7,9,13-15] To date, few clinical trials have focused on this group. Beyond lowering blood pressure and reducing the risk for stroke, the effectiveness of antihypertensives in reducing the progression of executive dysfunction remains unknown. The significance of studying the effect of antihypertensive therapy on executive function is based on these observations: only hypertensives with executive dysfunction, rather than memory decline, progressed to dementia;[16] hypertensive individuals with executive dysfunction have similar mortality to those with dementia;[17] and, as we have demonstrated in a previous report, hypertension with executive dysfunction has higher mortality than hypertension alone.[18]

2- *ACEI vs ARB in the brain*: There is a brain renin angiotensin system (RAS) that potentially links hypertension to cognitive function.[19-23] In the brain, Angiotensin II (Ang II) binds 2 main receptors, among others, AT1 and AT2 which have opposing effects. AT1 activation leads to vasoconstriction, endothelial dysfunction and smooth muscle hypertrophy. In animals, AT2 activation decreases infarct area after an ischemic injury by increasing cerebral perfusion in the penumbra,[24] decreases superoxide production,[24] activates neuronal repair systems by promoting neuronal cell differentiation and neurite growth, [25] and decreases inflammation and axonal degeneration.[26-29] AT_2 receptors are distributed in the cerebrovascular wall

and in the thalamus, hypothalamus and brain stem among other locations and AT2 activation may positively affect cognition.[22,30] ACEIs decrease Ang II production and hence decrease activation of both receptors whereas ARBs block AT1 but not AT2. ARBs also increase Ang II and possibly up-regulate AT_2.[31] This selective blockade of AT1 and possible hyperstimulation of AT2 may provide superior and brain-specific beneficial effects compared to ACEI.[32-34] Based on this AT2-hypothesis, we are suggesting that ARBs have superior effects relative to ACEIs in executive and cerebrovascular function. In the largest observational study to date, use of ARBs was associated with lower risk of dementia and Alzheimer's disease (AD) compared to ACEI and other antihypertensives.[11] In our pilot study, candesartan was superior to lisinopril in preserving executive function.

3- *Cerebral perfusion in hypertension and executive MCI*: Adequate cerebral perfusion is important for cognitive performance and hypertension is associated with declines in cerebral perfusion and cerebrovascular reserve.[35-37] Our recent work has shown that hypertensives have significantly decreased perfusion and reserve assessed by ASL-MRI.[38] Decline in perfusion is associated with executive dysfunction.[37,39,40] In our pilot study, we found that decreased cerebrovascular reserve, assessed by measuring the change in cerebral blood flow in response to carbon dioxide (CO2),[38] is associated with decreased executive function (see prelim data). Other studies suggest a decrease in CO2 vasoreactivity in patients with dementia.[41,42] Recent work by our collaborator's team suggests that executive MCI patients demonstrate hypoperfusion in the middle frontal cortex and precuneus.[40] RAS is involved in cerebral perfusion regulation.[43,44] We recently reported that genetic polymorphisms in RAS are associated with cerebrovascular reserve,[45] and others found an association of RAS genetic polymorphisms with vascular brain injury.[46,47] In animal studies, activation of AT2 improved cerebral perfusion and ARBs improved cerebral perfusion and ameliorated ischemic changes from atherosclerosis and hypoperfusion.[32,34,48,49] Since ACEI and ARB have differential effect on AT1 and AT2, we hypothesize that ARBs will be superior to ACEI in their effects on cerebral perfusion and reserve. Our preliminary data suggest that ARB use may be associated with improved cerebral blood flow velocity compared to ACEI.

4. *White matter integrity and executive function*: Hypertension and executive impairment are also associated with microvascular brain disease in similar overlapping regions and tracts (frontal and subcortical regions) notably presenting as white matter hyperintensities (WMH) on MRI or with loss of white matter integrity on diffusion tensor imaging (DTI) even in normally appearing white matter.[50-57] DTI-based indices of white matter integrity are very sensitive early indicators of vascular damage in the aging brain.[58] DTI also provides high sensitivity to detect white matter change over a period of 12 months.[59] New preliminary work by our team suggests that hypertension is associated with decreased Fractional Anisotropy in the frontal lobe. More recently, Diffusion Tensor Tractography (DTT) has been used to visualize and quantify integrity in specific brain networks and tracts.[60] Executive dysfunction is associated with loss of integrity in the left anterior cingulate tract[61] and precuneus.[62] The effect of antihypertensive therapy on white matter integrity has not been studied. Since AT2 activation is associated with axonal regeneration and improved neuronal repair,[63,64] ARBs may have superior effects on white matter integrity measures.

5. *Mechanistic approach for the effect of ARBs on the brain*: To further support our AT2 hypothesis in the brain, we will measure processes that are likely to be affected by AT2. AT2 activation is associated with decreases in vascular remodeling and inflammation and restores endothelial dysfunction by improving NO homeostasis.[28,30] Increased vascular stiffness and atherosclerosis reflected by higher carotid intima-media thickness (CIMT) contributes to vascular brain injury and cognitive impairment.[65-73] AT1 activation leads to greater atherosclerosis and vascular stiffness and ARBs, more so than ACEIs,[74] lower CIMT and arterial stiffness.[75-83] In the cerebral arteries, candesartan resulted in the normalization of mean media thickness, leading to decreased arterial stiffness.[84] ARBs also lower inflammatory markers, more so than ACEI,[85] and restore proper endothelial function possibly via AT2 hyperstimulation.[34,86] Inflammation also impairs endothelial function and increases atherosclerosis.[87,88] Recent evidence suggests that proper endothelial function requires the replacement of dysfunctional cells and that endothelial progenitor cells (EPC) function as a reservoir for newer endothelial cells.[89] The number of EPC has been linked with arterial stiffness, atherosclerosis, vascular brain injury and dementia.[90-92] ARBs may improve the proliferation and function of EPCs in hypertension via an antioxidant effect.[93] Therefore the potential mechanisms by which ARBs may prove to be superior to ACEIs in the brain may be related to their effects on atherosclerosis, vascular stiffness, inflammation, and optimal endothelial function and turnover. This project will test these hypotheses.

2.2 Study Rationale

Selective blockade of AT1 (ARB) vs dual blockade of AT1 and AT2 (ACEI) allows testing the AT2-hypothesis.[94,95] We suggest that the non-inhibition of AT2, AT2 up-regulation and increased Ang II that results from ARB treatment would explain this superior effect. AT1 and AT2 have opposing effects on endothelial function, inflammation, and vascular stiffness, which in turn are associated with perfusion, cerebrovascular reserve (CO_2 vasoreactivity), white matter connectivity and executive function. We will be able to test these hypotheses by comparing the effect of ACEI and ARB on executive function (AIM1), cerebrovascular function and white matter connectivity (AIM2), and investigate underlying vascular, endothelial, and inflammatory mediators (AIM3).

2.3 Prior Studies

1- *Safety and blood pressure trajectory of short-term withdrawal of antihypertensive medications in older adults*: The short-term safety of, and blood pressure changes after withdrawing hypertension treatment in older adults in preparation for clinical trials have not been well established. Participants were enrolled in a clinical trial and antihypertensive medications were tapered over three weeks (week 1: reduction by 25-50%, week 2: 50-75%, week 3: off). Blood pressure was measured at the initial visit and after stopping all antihypertensive therapy (personnel) and twice a day during the taper phase (provided monitor). Trend analyses and linear models were used to assess changes in blood pressure. All participants (n=53, mean age=71 years, total of 1158 readings) successfully tapered their medications with no symptoms. Only 2% of the readings exceeded 180/100 mm Hg but none were consecutive. Blood pressure gradually increased with an overall increase of 12/6 mm Hg, 95% confidence interval (CI) [4/1,21/11]. The daily increase in blood pressure was 0.2 mm Hg (SE=0.1) in both the systolic and diastolic blood pressure. Increases in systolic and diastolic blood pressure were comparable for all antihypertensive classes (p>0.05 for all). Blood pressure changes are displayed in **Figure 1**. Short-term (<3-4 weeks) withdrawal of antihypertensive therapy in older adults with hypertension is safe and is associated with mild increases in blood pressure.

Figure 1: Trajectory of daily blood pressure over time in the study participants. Solid lines are the mean blood pressure at each day and dotted lines are the 95% confidence interval

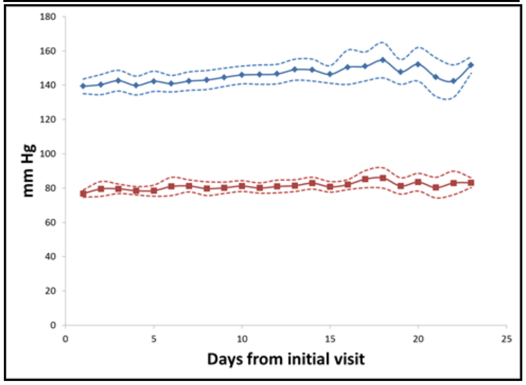

3. STUDY DESIGN

This is a double-blind randomized clinical trial in individuals with hypertension and executive MCI without dementia. Participants will be treated for 1-year with candesartan or lisinopril. Additional antihypertensives will be added to achieve blood pressure control to below 140/90 mm Hg in both groups. Our study will recruit 160 participants to achieve a sample size of 140 with 70 participants in each group. Our outcome measures include cognitive function, cerebral perfusion and reserve, markers of vascular brain damage, atherosclerosis, stiffness, vascular inflammation and endothelial function.

4. SELECTION AND ENROLLMENT OF PARTICIPANTS

4.1. Inclusion Criteria

(1) Age: 55 years or older;

(2) Hypertension: SBP≥140 mm Hg or DBP≥ 90 mm or receiving antihypertensive medications. Currently treated hypertensive individuals are eligible if they are willing to taper their medications. We will use a step-wise protocol of lowering antihypertensive medications;

(3) Executive MCI will be defined using these criteria:

(a) The Montreal Cognitive Assessment (MoCA) score less than or equal to 26: MoCA is a 30-point scale administered in 10 minutes, is validated in multiple languages and is highly sensitive to identify mild vascular-related cognitive dysfunction[96] (sensitivity 90% and specificity 87%).[97,98]

(b) Executive dysfunction[40,99,100]: A performance at the 10th percentile or below on at least one of four screening tests for executive function: Trail Making Test, Part B (TMT-B), modified Stroop interference, Digit Span and Digit Sequencing, and Letter fluency[100]

(c) Minimal Functional limitation as reflected by the Functional Assessment Questionnaire (FAQ)≤7 .

4.2 Exclusion Criteria

(1) Intolerance to candesartan or lisinopril ;
(2) SBP >200 or DBP >110 mm Hg;
(3) Renal disease or hyperkalemia (Serum Cr >1.99 mg/dl or K >5.5 meq/dl);
(4) Active medical or psychiatric problems (An active problem is one that requires medical attention and is deemed as a safety risk by the study physician, for example current cancer treatment, infections such as pneumonia, current gastrointestinal or other internal bleeding, HIV treatment, hematological disease, Parkinson's disease, multiple sclerosis)
(5) Uncontrolled congestive heart failure [shortness of breath at rest or evidence of pulmonary edema on exam);
(6) History of stroke in the past 3 years (self-reported clinical stroke within the past 3 years/ incidental infarcts on MRI scanning is allowed);
(7) Inability to perform the study procedures (those with contraindications for MRI will be enrolled but will not undergo the MRI; those with history of asthma, COPD, or significant pulmonary disease will not undergo the CO_2-reactivity protocol);
(8) Women of childbearing potential
(9) A diagnosis of dementia self-report or care-giver report
(10) In those who lack decision capacity, a *study surrogate* who can sign on their behalf will be required. Since we are enrolling only those with MCI, we anticipate that most participants will have decision capacity
(11) Current use of Lithium, as most antihypertensive classes may lead to increased lithium toxic levels.

4.3 Study Recruitment and Payment

Potential participants will be identified through community activities, health fairs, advertisements and mail out announcements. To recruit minorities, we will perform blood pressure screening activities in targeted community barbershops and beauticians, where information about the study will be provided. We have previously used barbershops and beauticians for recruitment of African Americans into a hypertension study with significant success. Below we describe our recruitment venues:

(i) The Emory Alzheimer's Disease Research Center (ADRC) Clinical Core registry of research participants will be utilized for recruitment. The registry includes about 400 individuals who have consented to participate in annual detailed research assessments with neuropsychological testing, neurological examination, and other components of a national uniform data set for the NIH designated Alzheimer's disease centers. This group of subject includes about healthy elderly individuals without cognitive symptoms or decline (controls, n=~100)), as well as subjects with mild symptoms and memory loss (mild cognitive impairment, n=~150), and Alzheimer's disease (n=~100). All subjects are interested and highly motivated to participate in research studies and have consented to be contacted.

(ii) Community-based recruitment: This will include announcement and recruitment information in periodicals and local newspapers. Blood pressure education sessions in local communities e.g. churches or barbershops will also be conducted.

(iii) Physician recruitment: Local physicians (primary care or specialty physicians) will be informed of the study and its requirements and provided information about referral to the study personnel. In addition, flyers for the study will be posted at outpatient areas in the following facilities: Emory University Hospital, Emory Clinic, Grady Memorial Hospital, and the VA medical Center if possible.

(iv) Study Visit Compensation:

- Screening visit with consent and cognitive testing only- $10 gift card.
- Screening visit with consent, cognitive testing, physical exam, and blood sample- $25 gift card.
- Baseline visit- $75 gift card, 3 and 6 month follow up visits- $25 gift card, 12 month follow up visit- $75 gift card.
- No compensation will be given during the blood pressure medication adjustment visits.
- Transportation Reimbursement: A parking validation ticket ($6 value) will be given to those subjects who drive and park at the Lowergate parking deck for the ACTSI portion of the study. Patients who require taxi service or are traveling by car from a long distance to the study visits can be reimbursed up to $50 each way (Total of $100 per visit). This payment will also be given in the form of gift cards.

5. STUDY INTERVENTIONS

5.1 Interventions, Administration, and Duration

Participants will be randomized into either candesartan or lisinopril treatment groups. Treatment will be provided in a capsule format to be taken once a day orally. Both candesartan and lisinopril will be formulated into identical capsules. Investigators, study personnel and participants will be blinded for drug assignment. Participants will be treated for 1 year.

5.2 Handling of Study Interventions

The drugs will be stored in the medication room in the Investigational Drug Service (IDS) Pharmacy. The location of the IDS pharmacy is The Emory Clinic Bldg. A, Suite 1200, 1365 Clifton Road, NE, Atlanta, Georgia 30322. Access to the med room is limited to the IDS Pharmacists via a badge swipe. Accountability records are maintained for all investigational products (IP). Study medication bottles will be either delivered by courier to study site, get picked up by study personnel from the pharmacy, hand delivered to subjects, or mailed to subject's home. Subjects will receive 30, 45, or 90 day supply. At the completion of the study, each participant will be unblinded and given at least a 30 day supply of the study medication. Unblinding will occur at the participants 12 month F/U visit and PCP will be notified of the study drug and trial completion. The PI/investigators will remain blinded to the treatment)

5.3 Concomitant Interventions

The goal of the trial intervention is to achieve a blood pressure <140/90 mm Hg in at least 90% of the participants. To achieve blood pressure control, we will use a stepwise protocol as follows: candesartan 8 mg→16 mg→32 mg or lisinopril 10 mg→20 mg→40 mg. Both groups will also receive, if needed to achieve blood pressure control, HCTZ 12.5 mg→25 mg, Amlodipine 2.5 mg →5 mg→10 mg and metoprolol 12.5 mg→25 mg (extended release)→50 mg (extended release). These will be added in a stepwise and escalating fashion so that each new as needed drug will be added to the prior medication regimen, unless noted by the PI. Antihypertensive medications will be increased every 2 weeks until blood pressure control (less than 140/90 mm Hg) is achieved. Based on our prior study, we anticipate being able to achieve blood pressure control within 4-8 weeks in the majority of the participants. Only the candesartan and lisinopril treatments are blinded. The remaining treatments (HCTZ, Amlodipine, and Metoprolol) are open-label. In the instance where a participant cannot receive an add-on agent, e.g. if there is a history of allergy or intolerance to one of the add-on drugs (HCTZ, Amlodipine, and Metoprolol) or a contraindication due to drug-drug interaction (e.g. simvastatin dose >20 mg and amlodipine) or other medical safety reasons, then that add-on agent will be skipped and the next add-on drug will be used.

5.4 Allowed Interventions

Participants will continue to receive their usual care from their regular primary physicians. Hypertension management will be addressed by the study physician (the PI). In the event that a participant's potassium level decreases during the study, a prescription for potassium will be provided to the subject as clinically indicated by the physician.

5.5 Required Interventions

In addition to the 2 study medications (candesartan and lisinopril), HCTZ, amlodipine, and metoprolol are allowed as described above to achieve blood pressure control to below 140/90 mm Hg. Medications will be adjusted up or down to maintain blood pressure control (<140/90 mm Hg) during the study period.

5.6 Prohibited Interventions

Once participants are enrolled, addition of antihypertensive medications by non-study providers is not allowed. However, non-hypertensive medications are allowed as part of usual care. If a subject receives an antihypertensive medication from a non-study source after enrollment, the study physician will attempt to contact the prescribing provider, as long as the participant agrees, to explain the study protocol and request that all antihypertensive medications be prescribed through the study physician. If the participant or the provider does not agree to these recommendations, the participant will be withdrawn from the study for safety reasons.

5.7 Adherence Assessment

Participants will be asked to bring their study medication bottles to the study center at each visit. Medication compliance will be assessed using pill count during the titration and follow up periods. We will define a compliance rate for a time period, t, as the ratio of: (the used number of pill prescribed for the number of days t- number of pills remaining or unused for the time t/ number of pills prescribed for time t) multiplied by 100.

6. EXPERIMENTAL PROCEDURES AND MEASURES

6.1 Questionnaires and lifestyle and anthropometric measures

Study interviews will be conducted in English as necessary and include the following:

(1) Demographic, social and medical history data, as well as a medication inventory will be collected. All participants are asked to bring all their prescribed medication bottles. The medication inventory will be done by inspecting the participant medication and recording the name, dose, frequency and indication if known.[101]

(2) Instrumental activities of daily living (IADL) scale .[102]

(3) We will specifically evaluate functional abilities related to the executive cognitive function using the Dysexecutive Functioning Questionnaire (DEX). [103,104] DEX is a 20-item questionnaire, which is completed by the participant and study partner and is designed to assess everyday signs of executive difficulties.

(4) Weight and height (stadiometer to measure height with the subjects standing and balance beam scale to measure weight without shoes).

(5) Short Physical Performance Battery [SPPB: ability to stand with the feet side-by-side, semi-tandem, and tandem, time to walk 8 feet (measured twice), and time to rise from a chair and return to the seated position 5 times) which assesses balance, gait, strength of the lower extremities, and endurance in older persons];[105]

(6) Physical activity will be assessed using the Physical Activity Scale for the Elderly (PASE), which measures occupational, household, and leisure activities during a one-week period and has high reliability in older adults.[106,107]

(7) Two Perceived discrimination scales will also be collected once at screening or during the study Follow-up to account for the level of stress in the participants with hypertension.

6.2. Blood pressure measurement

(1) Office blood pressure will be measured according to the American Heart Association guidelines: sitting position, rested for 5 minutes, appropriate cuff size (covering 60% of upper arm length and 80% of arm circumference), correct cuff placement (1-2 inches above brachial pulse on bare arm), and use of the bell of the stethoscope.[108] Blood pressure will be measured in both arms. The arm with the higher blood pressure will be used throughout the study. We will obtain 2 seated followed by standing blood pressure measurements at 1 and 3 minutes during each visit.

6.3 Neuropsychological assessment:

(i) Executive function will be assessed using Trail Making Test, Part A, B and B-A

(ii) EXecutive Abilities: Measures and Instruments for Neurobehavioral Evaluation and Research or "EXAMINER" tool box. This test battery reliably and validly assesses multiple subdomains of executive function and related areas.[109-111] The battery includes 11 tasks that generate 15 primary variables. Within this set, the EXAMINER includes: working memory, inhibition, set shifting, and fluency. The parts of EXAMINER that we selected include:

1) Flanker task (inhibition) which involves responding to a central stimulus while ignoring flanking stimuli that are either compatible or incompatible with the central stimulus.[112]
2) Set-shifting, a measure of mental flexibility assessing the subject's ability to attend to the specific attributes of compound stimuli, and to shift that attention when required.[113]
3) Spatial 1-Back test assesses spatial working memory and
4) Dot Counting test assesses verbal working memory.[114]
5) Verbal Fluency will be tested using a List Generation test which require the participant to generate words beginning with a specific letter, and category fluency in which the participant generates words from a specified category (e.g., animals, fruits).[115]

(iii) Hopkins Verbal Learning Test will be used to assess memory domains.
(iv) Digit Span Test (DST) is a brief task that assesses attention.[116,117]
(v) Boston Naming Test assesses language by measuring ability of naming a visual confrontation drawing (15 items).[118,119]
(vi) Center for Epidemiologic Studies Depression Scale (CESD),[120] and consists of 20 items, each scored from 0 to 3 points and higher scores indicate greater depressive symptoms.

6.4 Brain MRI imaging protocols

MRI protocols are performed in 50-60 minutes and will be conducted at The Center for Systems Imaging (CSI). Table 1 provides the details of the imaging protocol.

Table 1: Neuroimaging sequences and scanner time

	Orient.	Voxel Size [mm]	Scan Time [min]	
Localizer			29 sec	
T1 MPRAGE	Sagittal	1.0x1.0x1.0	8 min 37sec	
3D T2 TSE SAG SPACE	Sagittal	1.0x1.0x1.0	6 min 58 sec	
BOLD CVR	Axial	3x3x3	6 min 9 sec	1off-1on-1off-1on-2 off C02
Remove Mask				
3D T2 FLAIR SPACE	Sagittal	1.0x1.0x1.0	7 min 59 sec	
2dPCASL	Axial	3.4x3.4x5 (1mm gap)	6min 11 sec	
DTI Axials	Axial	2x2x2	4 min 52 sec	30 directions
rs-fMRI	Axial	3.0x3.0x3.0	8 min 9 sec	
GRE Field Map	Axial	3.0x3.0x3.0	1 min 20 secs	
Neck Coil scans for labeling efficiency				
3D TOF MRA Carotids	Axial obliq	0.6x0.6x1.5	1 min 21 sec	
2D Phase Contrast	Axial obliq	0.7x0.7x5	27 sec	
**Total Scan Time**			**53**	

6.5 Vascular measures

(i) Carotid intima-media thickness and arterial stiffness: High-resolution B-mode ultrasonograms of the right common carotid artery will be obtained using a 7.5-MHz linear-array transducer attached to a digital ultrasound system. Participants are placed in a supine position with the head rotated to the left by using a 45-degree head block. Longitudinal images of the common carotid will be obtained. Indices of arterial stiffness and wave reflections will be estimated in the supine position using the Sphygmocor device (Atcor Medical, Australia), which records sequential high-quality pressure waveforms at peripheral pulse sites using a high-fidelity tonometer. Pulse-wave velocity (PWV) measured between carotid and femoral arteries is a regional assessment of aortic stiffness and is the gold standard index of arterial stiffness. Digital pulse amplitude tonometry (PAT) will be used to measure pulse volume amplitude (PVA) in the tip of the index finger, with participants resting in the supine position in a quiet, temperature-controlled environment and during reactive hyperemia, which will be elicited by the release of an upper arm blood pressure cuff inflated to suprasystolic pressure for 5 minutes. The Endo-PAT (Itamar-Medical, Israel) will be used to measure PAT. The reactive hyperemia index (RHI) will be calculated as the ratio of the post- to pre-occlusion pulse volume amplitude (PVA) of the tested arm, divided by the post- to preocclusion ratio of the control arm (the average PVA over a 1-minute interval starting 1-minute after cuff deflation divided by the average PVA measured for 1 minute before cuff inflation [baseline]).

(ii) Inflammation, endothelial function markers, and APOE: We selected inflammatory biomarkers based on the evidence that the marker may be associated with cognition and/or is affected by RAS/ARB. Various studies have suggested that these inflammatory markers are associated with cognitive function, increased in hypertension, and modulated by Ang II: C-reactive protein (CRP)[85,121], cytokines (TNF-α, interleukin family),[85,121] vascular adhesion molecules (ICAM, VCAM, E- and P-selectin)[122-125], metalloprotease 9 (MMP9)[85] fibrinogen, von Willebrand factor (vWF) and plasminogen activator inhibitor-1 activity (PAI-1).[121,126-128] Endothelial progenitor cells (EPC) will be measured according to methods previously described.[91] Mononuclear cells will be first isolated by density gradient from 10 cc of blood. EPC will be identified by the expression of surface antigens CD34+KDR+ which provides the best balance between cell count and accuracy (ICC=0.81, CV<10%),[129] and reported as the number of cells per cytometric event to avoid error inflation due to variation in total blood cells. [91,130] We will use EPC and adhesion molecules to further investigate the association between cerebrovascular reactivity and endothelial function. EPC measurement will be completed at ECCRI (Dr. Waller's Lab) at Emory. We will perform baseline, 12 months assessments of the inflammatory biomarkers and baseline, 6 and 12 months assessments of the EPC and urine chemistries. Blood samples will be drawn at baseline for APOE genotype as well.

6.6 Blood chemistries and blood count,

Blood chemistries (metabolic panels) will be measured at various phases of the study including but not limited to screening, baseline and during the follow-up as part of the screening and safety monitoring for hyperkalemia and renal insufficiency, as shown in Table 2. A complete blood count will also be collected at baseline and 6, and 12 months. Additional blood tests can be drawn at the discretion of the physician if needed for safety, eligibility confirmation, or follow-up on abnormal findings. Blood will be drawn after application of a tourniquet, by use of a small gauge needle, from an ante-cubital vein, and by an appropriately trained professional from subjects. These are part of assessing safety of the study drug and will be performed at the local Emory Clinical Laboratory.

7. STUDY SCHEDULE

7.1 Schedule of Evaluations

The flow of the participants from screening to exit is described diagrammatically in figure 4 and the list of study procedures performed at each visit is detailed in Table 2.

Table 2: Study procedures at various stages of the study

Phase:	Screening/Enrollment		Baseline	Titration	Follow-up		
**Number of visits**	Phone	1	1	3-5	3		
**Months**	0	2-4w	1	2-3	3	6	12
**Informed Consent**		X					
**Study forms**	Screen1	Screen2	Baseline	FU-tit	FU3	FU6	FU12
**UBACC**		X					
**Phone contact/frequency***	X	W			M	M	M
**Screening Cognition and FAQ**		X					
**Letter to PMD**		X					X
**Antihypertensive taper**		X					
**H&P****		X	X	X			
**Medication inventory**		X	X	X	X	X	X
**Weight**			X	X	X	X	X
**Height**			X				X
**Blood pressure**		X	X	X	X	X	X
**Metabolic Panel *****		X	X	X	X	X	X
**CBC**			X			X	X
**IADL, SPPB, DEX , CESD**			X				X
**Neuropsychological measures**			X			X	X
**Perceived discrimination******	X	X		X	X		
**Brain MRI**			X				X
**Vascular imaging**			X				X
**APOE**			X				
**EPC & Urine Chemistries**			X			X	X
**Inflammatory markers**			X			X	X
**Screening for AE/SAE**				X	X	X	X
**Time estimate, in minutes**	15	40-60	210-240	20-30	20-30	60-90	210-240

*: Frequency is at least once or twice per week (W) during tapering pre-visit antihypertensive medications and Monthly (M) during the FU phase.
**: A History and physical may be performed at screening, baseline or titration/FU phase
***: Labs will be drawn at specified times as in Table 2 (screening and/or baseline, titration, and FU) and at additional times if necessary, for eligibility confirmation, follow-up on abnormal labs, or for additional safety measures. If BMP is drawn at screening then only Liver Function Test/Liver Profile code at EML 1003193 will

be drawn at Baseline to avoid duplicate labs. If BMP was not drawn at baseline, then a comprehensive metabolic panel will be drawn at Baseline.
****: This scale will be collected once at screening, baseline or during the follow-up.

Study Visit Windows Table:

**Study Visit Windows TABLE 3**	
**Baseline**	Within 1 week of completed tapering; patient should not be off BP meds for longer than 1 week.
**Med adjustment/Titration**	+/- 3 days of 2 week visit after BL
**Med adjustment/Titration**	If blood pressure is not regulated at first titration visit, must return +/- 3 days of next 2 week visit- return every 2 weeks until BP is regulated.
**3 month F/U**	+/- 2 weeks of scheduled visit
**6 month F/U**	+/- 2 weeks of scheduled visit
**12 month F/U**	+/- 2 weeks of scheduled visit

7.2 Description of Evaluations

7.2.1 <u>Screening</u>: Initial eligibility will be determined via phone interview. This screening process will determine the specific inclusion and exclusion criteria that can be evaluated by self-report, including age, hypertension or blood pressure diagnosis, and prior medical issues detailed in the inclusion/exclusion criteria. Data collected during this interview will be stored de-identified for future data analysis with no links to the patient or their PHI. A study ID number will be used to collect data and will not be linked to the participant. Those who are eligible via phone interview will be invited for a clinic screening visit along with their next-of-kin, if possible. The screening visit will include: explaining study details and obtaining informed consent, cognitive assessment, medical history, current medications, 2 seated blood pressure measurements, and baseline metabolic panel. Eligible participants currently receiving antihypertensive medications who agree to stop antihypertensive medications will be instructed on a taper protocol and asked to return for baseline evaluation and randomization.

7.2.2 <u>Consenting Procedure</u>: A signed consent form will be obtained from each participant or the participant’s legally authorized representative. A single informed consent form will describe both the screening and study procedures. The consent form will describe the purpose of the study, the procedures to be followed, and the risks and benefits of participation. A copy will be given to each participant and this fact will be documented in the participant’s study record and the participant’s medical record as per Emory University policy. During the consenting process, an assessment for decisional capacity to participate in clinical research will be performed. A validated brief instrument for decisional capacity assessment will be administered:[131] The University of California, San Diego Brief Assessment of Capacity to Consent (UBACC) instrument is a 10 item questionnaire that asks the participant about key areas of the informed consent. **A score greater than 14.5** correlates with a core of greater than 16 on the MacArthur Competency Assessment Tool for Clinical Research (MacCAT-CR).[131] MacCAT-CR is the most validated instrument in assessing decisional capacity in clinical research[132] and a score greater than 16 has been traditionally considered adequate in prior NIH studies.[133] Participants who are deemed non-decisional but still qualify for the study will need to have a surrogate consistent with the intent of the Common Rule (45 CFR 46, Subpart A).The following are, in order, possible surrogates:

(1) The person's agent designated by an advance health care directive.
(2) The conservator or guardian of the person having the authority to make health care decisions for the person.
(3) The spouse of the person.

(4) The domestic partner of the person as defined in Section 297 of the Family Code
(5) An adult son or daughter of the person.
(6) A custodial parent of the person.
(7) Any adult brother or sister of the person.
(8) Any adult grandchild of the person.
(9) An available adult relative with the closest degree of kinship to the person.

When there are two or more available persons who are in different orders of priority pursuant to subdivision (c), refusal to consent by a person who is a higher priority surrogate shall not be superseded by the consent of a person who is a lower priority surrogate.

7.2.3 Enrollment, Baseline, and Randomization: During this phase, blood pressure measurements, detailed neuropsychological assessments, brain MRI, and carotid ultrasound will be completed. Blood samples will also be collected for APOE, inflammatory and endothelial markers. Randomization and study intervention will occur after baseline data collection. Participants will then be started on the study medications. For all study related testing, the test results will not be disclosed to participants unless deemed necessary by the study Physician.

7.2.4 Follow-up Visits: Following randomization, participants will be seen every two weeks, where antihypertensive study medications will be increased until they achieve blood pressure control (<140/90 mmHg), according to the designated trial protocol for blood pressure control. Blood pressure, heart rate, weight, adverse events (AE) and serious adverse events (SAE), pill count (to assess compliance), and use of non-study medications data will be collected. Once hypertension control is achieved, participants will be seen at 3, 6 and 12 months visits. Repeat neuropsychological assessment, blood draws (EPC and inflammatory markers), and urine collection will be conducted at the 6 and 12 months visit. Repeat neuroimaging and vascular ultrasound will be repeated at 12 months. BMP will also be checked at 2 weeks after randomization if only step one is needed, 4 weeks post randomization if treatment beyond step 1 is needed, and at 3, 6 and 12 months as shown in **Table 2**. Participants will also be contacted by phone as described in Table 2, monthly during the follow-up phase. During these phone calls, participants will be asked about missing any doses in their medications and screened for any potential adverse events. If participants are titrating during the first and second monthly phone calls, the titration visits will be used instead of these phone calls to obtain this information.

8. SAFETY ASSESSMENTS

All data derived from this study is for research purposes only. Subjects will be monitored at all times for adverse events.

8.1 Potential Risks

The proposed study will utilize widely used and approved procedures including: commonly used neuropsychological questionnaires and interview materials, blood pressure measurements, brain MRI, carotid ultrasound, and blood draws. Neuropsychological assessment may be accompanied by anxiety, frustration and overall fatigue. The attachment and removal of a blood pressure cuff, cuffs for venous occlusion, and ultrasound probe on the neck may cause mild discomfort. Brain imaging requires the participant to stay still and lie down for 50-60 minutes, which may cause boredom and minimal reversible back pain. Because of the closed space and noise, undergoing an MRI may be associated with anxiety or panic reactions. Claustrophobic-prone individuals will not be asked to undergo the MRI portion. We will ask participants to breathe via a mask air richer in CO_2 than normal atmospheric air (5-8%) during the brain MRI procedure. The CO_2 reactivity procedures were well tolerated by elderly subjects in our prior studies, and have been performed numerous times in our laboratory without complications. The reported potential side effects may include a feeling of dizziness, faintness, or anxiety during CO_2 inhalation.

The discontinuation of blood pressure medications may be associated with a risk of marked blood pressure elevation and development of associated neurological or cardiac symptoms: headaches, dizziness,

blurred vision, chest pain or ischemic neurological events. In our pilot study, none of the participants experienced these symptoms. To minimize this risk, medications will be slowly tapered over a 2-4 week period while study personnel carefully monitor subjects.

The proposed study will utilize drugs approved by the Food and Drug Administration for the treatment of hypertension. They are well tolerated by young and elderly individuals and have been used extensively in clinical practice. Possible side effects include: dizziness, cough (lisinopril), weakness, fatigue, lower extremity edema and constipation (amlodipine), and hypotension. Renal failure and hyperkalemia are also potential complications of candesartan and lisinopril. HCTZ may cause renal failure, volume depletion, and hypokalemia. Lisinopril may cause a dry cough and angioedema. Beta blockers may be associated with weakness and bradycardia. Adverse events will be queried at each visit. Alternatives to antihypertensive medications include no therapy, which is not safe or ethical in patients with hypertension. Drug-drug interaction will be assessed by the study physician. If there is contraindication to use the candesartan or lisinopril and one of the other participant's medications then he/she will not be able to enroll in the study. If the contraindication is between an add-on agent (amlodipine, metoprolol, or HCTZ) then the participant will be enrolled but we will avoid using that agent as an add-on as described in section 5.3. Use of lithium is a contraindication for both lisinopril and candesartan and hence lithium users are excluded. The following combinations of medications are contraindicated for the add-on drugs: Simvastatin >20 mg with amlodipine, reserpine with metoprolol, and clonidine with metoprolol.

8.2 Specification of Safety Parameters

The study medications may lead to adverse drug reactions (rash, edema, and in severe cases anaphylaxis) and uncomfortable side effects. If this occurs, the study medication will be discontinued and the subject's primary care provider notified. If the subject cannot tolerate the study medication, the study medication will be stopped and he/she will be referred to their primary care physician for ongoing hypertension treatment. The participant will however be continued in the study and asked to undergo the final visit evaluations. Subjects will be monitored carefully for symptoms, blood pressure control, and blood chemistry abnormalities during treatment, as described in the methods section above. A physician (PI) and trained personnel will be available at all times to respond to subjects' concerns. In the event of an emergency, resuscitation equipment is readily available in all the research areas where subjects are evaluated. Antihypertensive medications will also be adjusted up/down to maintain blood pressure below 140/90 mm Hg and above 100/45 mm Hg or at the discretion of the study physician for maintaining safety. For example, if blood pressure readings are high at their study visits but normal/low at home or the subject reports orthostatic dizziness the clinician will use his judgment to change the dosage of the study meds.

The subject's primary care physician will be informed of study plans. Frequent monitoring will include visits, blood pressure checks, laboratory testing and phone contact. Blood testing for kidney function and electrolytes (potassium) will be performed at baseline, 1, 3, 6 and 12 months. If needed, additional calls, visits, or laboratory testing will be conducted as decided by the PI and /or the primary care physician. If symptoms develop during any of the evaluations, procedures will be terminated immediately. The experimental procedures will be conducted in a well-supervised facility and subjects will be under constant observation by skilled professionals. Those who could not continue on the study medications will be asked to return for collection of outcome measure if they agree to return for the final assessment visit.

(i) <u>CO2 Administration</u>: CO2 will be given via mask connected to a tank at a concentration of 5-8% and will be mixed with air and/or oxygen and nitrogen. To minimize respiratory discomfort, CO2 breathing intervals will be kept as short as possible to achieve desired increase in ETCO2 and will never exceed 2 minutes in length (continuous 2-minutes or two 1-minute blocks interspaced with 1-minute off). Heart rate, blood pressure, oxygen levels, and ETCO2 will be continuously and non-invasively monitored in order to limit risk for insufficient oxygenation, hypertension, and significant distress. In addition, the MR gantry is equipped with a camera/Glass window allowing continuous monitoring of the subjects from the MR control room; subjects are given a pneumatic bulb that starts off an alarm in the control room in case of emergency. CO2 administration will be promptly terminated if any of the following conditions occur: any complaint of subject discomfort, appearance of new neurologic symptom, systolic blood pressure increase of >50-55 mmHg or mean arterial

blood pressure increase of >35-40 mmHg over normo-capnic levels, or pulse acceleration over baseline of 30-40 beats per minute. To further minimize risk of complications, subjects with **active asthma or chronic obstructive pulmonary disease, will not be given CO_2.**

(ii) Neuroimaging: Should subjects feel discomfort during the MRI procedure the experiment will be terminated upon their request. The operators of the MRI scanner will take steps such as using foam pads when necessary to minimize the risk of excessive heating or burns from contact with the MRI transmitting and receiving coil or conductive materials. Subjects will be screened for magnetic material before each study, per the standard of care at the MRI suite. Patients with pacemakers, aneurysm clips, metallic prostheses or shrapnel fragments are at risk at an MR environment and will be excluded from the neuroimaging portion of the study. Women of childbearing age will not be included. Earplugs or close fitting silicon padded headphones will be provided to protect from damage to the inner ear due to the loud tone produced by the MRI scanner. Subjects will be observed and monitored at all times by the operators, and can be moved out of the machine at their request. Subjects will be able to communicate through an intercom with the physician/scientist or technician at all times. In addition the MR gantry is equipped with a camera allowing continuous monitoring of the subjects from the MR control room; subjects are given a pneumatic bulb that starts off an alarm in the control room in case of emergency. All people entering the exam room where the magnet is located will be screened for magnetic material before entering the room. This will apply to subjects, experimenters, and staff.

(iii) Incident dementia: Although limited information is available, it is estimated that the yearly conversion rate is close to 7% in executive MCI.[134] We will screen at each visit by asking the participant or/and study partner for a new diagnosis of dementia or the use of cholinesterase inhibitors or memantine. A yes answer to these questions will trigger a conversion to dementia assessment. . The physician (PI) will review the clinical history with the other investigators/clinicians to achieve investigators' consensus on conversion to dementia. . We will compare the number of people who convert to dementia in both groups as part of our safety analyses.

8.3 Antihypertensive replacement protocol

Those with hypertension who are receiving antihypertensive medications will have their current medications tapered gradually using an established protocol that has minimal risk. Their primary care provider will also be notified, if the participant agrees, before initiating any changes in the participant's blood pressure medications. If the subject agrees then a letter will be faxed or mailed to their primary care physician providing information about contacting the study personnel regarding any concerns or comments about the antihypertensive replacement. Participants will be provided with an automated blood pressure monitor and a blood pressure diary (Home Blood Pressure Monitoring Flow Sheet) to monitor their blood pressures at home. Contact by the study personnel will be weekly, or more if needed, for review of blood pressures. Once the participant is off all antihypertensive medications, he or she will undergo the baseline evaluation. The following protocol will be used for the withdrawal:

1- All participants will receive personalized written instructions on tapering and discontinuation of antihypertensive medications, description of symptoms associated with possible adverse events and familiarization with the study protocol (Home Blood Pressure Monitoring Flow Sheet). They will be instructed to share this information with their spouses or other members of their households. The timeline for medication tapering is summarized in the following table:

Number of antihypertensive meds	*Week 1*	*Week 2*	*Week 3*	*Week 4*
	Day 0-7	*Day 8-14*	*Day 15-21*	*Day 22-28*
One antihypertensive med	Half weekly dose	No meds<br>**Baseline Visit**		
2 antihypertensive meds	Half weekly dose med 1<br>Full weekly dose med 2	No med 1<br>Half weekly dose med 2	No meds<br>**Baseline visit**	

3 antihypertensive meds	Half weekly dose med 1<br>Full weekly dose med 2 and 3	No med 1<br>Half weekly dose med 2<br>Full weekly dose med 3	No med 1 and 2<br>Half weekly dose med 3	No Meds<br>**Baseline visit**

2- Subjects and/or proxy will be provided with a portable automated blood pressure (BP) monitor and a diary to take home. They will be asked to measure BP 2x times a day (morning and before sleep) and record into a diary.

3-Daily/weekly dose reductions are shown in the Table above. Baseline visit will performed after all antihypertensive medications have been stopped. In some cases when safety is a concern, the study physician may recommend not to taper certain blood pressure medications. Such situations include but are not limited to beta blockers or non-dihydropyridine calcium channel blockers (diltiazem or verapamil) in those with heart disease, atrial fibrillation or history of palpitations or irregular heart rhythm, diuretics in those with edema, or any other blood pressure medication used for non-hypertension indication. In the case that blood pressure medication was not tapered, then the subject will not be prescribed the corresponding blood pressure medication as part of the study protocol. For example, if the subject was receiving propranolol for headaches and hypertension, then propranolol will not be tapered and metoprolol will not be prescribed for this subject. ACEI and ARBs will always be tapered.

4- A summary of symptoms will be provided to the subjects and/or their proxy. They will be asked to share this information with their spouses or other family members living in the same household or other close caregiver. They will be instructed to call the investigators immediately if any of these symptoms occur to arrange the transfer to the nearest emergency room. They will be provided with a pager number for the investigators. Weekly contact with the subject will be conducted by phone to obtain the blood pressure readings and review of any symptom that occurred.

5- The subjects will be asked to repeat the blood pressure measurement within 60 minutes if the reading is above 180/110 mm HG and call the investigators (by phone or pager) anytime that they have two consecutive readings exceeding 180 mm Hg systolic or 110 mm Hg diastolic or have any unexpected discomfort, dizziness, or headaches. The study physician will then recommend based on the prior blood pressure readings and the participant symptoms to either alter the way the blood pressure medication is being reduced, take extra dose of the blood pressure medication, or resume all usual doses of all blood pressure medications immediately (the doses before the study) and continue measuring blood pressure as instructed. A failed taper is defined as persistent (>2 consecutive readings on 2 consecutive days) elevation in blood pressure >180/110 mm Hg. In the event that the participant failed the taper, the participant is asked to go back on their usual antihypertensive medications and is withdrawn from the study. The study personnel will contact the subject monthly for 2 months and they will be referred to their primary care physician for chronic blood pressure management. For their safety they will not be included in the study.

8.4 Methods and Timing for Assessing, Recording, and Analyzing Safety Parameters

At each encounter, standardized adverse event (AE) monitoring questions will be asked. At any point during the study period an adverse event is identified, it will be recorded in the participant research record along with the time/place of the event. Clinically significant abnormal laboratory results will also be recorded as adverse event in the subject chart. The study physician/nurse will be notified when an AE is identified. The adverse event is reviewed and if needed the participant is evaluated either by phone (if participant is not on site) or face-to-face (if participant is on site). Further evaluation maybe performed such as scheduling additional visits, further lab work, exam, or referral to the emergency department. AE's will be compared yearly between the 2 treatment groups and reported to the DSMB during the board's yearly meeting.

8.5 Adverse Events and Serious Adverse Events identification and reporting

During the screening process, if the participant is identified to have a problem requiring medical attention such as severely elevated blood pressure, they are referred to their primary care physician or the emergency room. The primary care physician will be notified of screening results by mail upon the participant request.

Adverse events and serious adverse events that occur after enrollment will be recorded in the participant chart and rated on the following scales: serious (YES/NO), related to the research (related, possibly related, probably related, unrelated, or unknown); severity (mild, moderate, severe, life-threatening, or death), and anticipated (YES/NO). When indicated adverse events will be reported to the Emory IRB, the data safety monitoring board (DSMB) and the NIH funding agency following the guidelines set forth by the Emory IRB reporting guidelines either promptly or during continuing review. Promptly reportable events (unanticipated problems or UP as defined by the Emory IRB reporting guidelines) will be reported within 10 days of their discovery by the study personnel/PI.

Participants who drop-out form the study due to adverse event will be referred back to their primary care physician for ongoing hypertension care.

8.6 Incidental findings

Incidental clinically significant blood analysis results, brain imaging, and carotid ultrasound findings will be reported to the subject and primary care provider if the subject agrees for further evaluation and management. In the unlikely event that an incidental finding requires immediate attention urgent care will be provided in a facility of the participant's preference.

8.7 Follow-up for Adverse Events

With the permission from the participant, we will contact the participant's primary care doctor and help him/her get medical follow-up for incidental findings. Adverse events that require follow-up such as increased potassium or increased creatinine will be monitored until resolved or are further followed by the primary care provider. If the event requires intervention discontinuation (see section 9 below), the participant will be transitioned to his/her usual antihypertensive medication(s) and referred back to the primary care provider for future hypertension management. They will be followed for 2 months post-withdrawal with a monthly phone call. They will also be invited to perform the final visit evaluation including all study procedures planned for that visit if the participant has been in the study more than 5 month post baseline evaluation.

8.8 Safety Monitoring

Safety procedures will be implemented in accordance with NIH safety policies for clinical trials. The safety monitoring will be conducted by the PI and a Data and Safety Monitoring Board (DSMB). The DSMB is comprised of 4 members (see section 14) .

Once a year, the DSMB will meet to review progress of the trial (recruitment and follow-up, protocol violations), assess the safety of the protocol, and address any issue that limits the study success. A report template will be drafted prior to initiation of recruitment. The report will include tabulation by treatment group of all AE and SAEs to be reviewed by the DSMB. If concerns arise about safety issues, the DSMB may request additional data and propose specific analyses. A report summarizing the discussions and recommendations for continuation of the trial will be generated from the DSMB meeting and provided to the IRB and NIH funding institute.

9. INTERVENTION DISCONTINUATION

A subject may choose to withdraw from the study for any reason. The investigators may also request that the subject withdraw from the study, for safety or other reasons. Withdrawn participants will continue to be

monitored by the study personnel for 2 months after the event if possible and invited to complete the final visit evaluation. The criteria for discontinuing a subject's participation include:

(1) the subject's request,
(2) serious adverse events that requires un-blinding,
(3) new stroke, transient ischemic attack, or myocardial infarction that limits ability to complete the study
(4) anaphylaxis or allergic reaction to study medications,
(5) disturbing and persistent cough (transient mild cough is not a discontinuation criterion unless participant requested it),
(6) inability to participate due to relocation or other personal reasons,
(7) renal failure (increase in serum Creatinine above 2.5 mg/dl) or hyperkalemia (greater than 5.8 meq)

10. STATISTICAL CONSIDERATIONS

10.1 General Design

The study is a 2-arm double-blinded RCT. The statistical analysis will follow the intention-to-treat approach. The analyses will test our hypotheses that subjects on Candesartan will show: (1) less cognitive decline in the executive domain and its subdomains, measured by Trail Making Test and the EXAMINER score, (2) greater cerebral perfusion and lower cerebral vasoreactivity and white matter hyperintensities, and (3) greater measures of brain connectivity, compared to subjects on lisinopril at 12 months. Our second set of hypotheses are that relative to lisinopril, treatment with candesartan is associated with (1) lower CIMT, and arterial stiffness, and (2) lower inflammatory and higher endothelial function markers at 12 months.

10.2 Randomization

The study un-blinded biostatistician will provide oversight of randomization fidelity and blinding. To ensure equal distribution between the 2 groups on important confounders, we will use block randomization with stratification on:

(1) race, (white, non-white).
(2) number of antihypertensive medications (2 or less vs >2).

Randomization will occur in a 1:1 allocation. Four separate randomization lists will be prepared (race=white, medications=2 or less; race=white, medications>2; race-non-white, medications=2 or less; race=non-white, medications>2). Randomization will use a computerized random number generator (SAS, V9.3); the blocking number will not be revealed to investigators or research staff. Each randomization list will include the treatment group allocation, a unique allocation sequence number, and a blank field (to complete with subject study ID as randomization occurs). Randomization lists will be provided to the IDS Pharmacy. Only the pharmacy and the un-blinded statistician will have access to the randomization lists. As the pharmacist is notified that an individual subject is eligible for randomization to a given stratum, the pharmacist will select the appropriate randomization list, complete the subject ID, and prepare the appropriate blinded study product. The pharmacist will enter the subject ID, date of product randomization, and the unique allocation sequence number on a web-based data form. The statistician will monitor appropriateness of randomization (eligibility criteria met, correct stratum used, next allocation sequence used) in real time; incorrect randomizations will be immediately recorded as protocol deviations and reported to the principal investigator (in a blinded fashion).

10.3 Interim analyses

There will be planned regular reporting to the DSMB of trial conduct and safety (see Data and Safety Monitoring Plan). We will conduct an interim outcome analyses for efficacy when 50% of the sample has completed their 12-month assessments in addition the final analysis. We will use an alpha spending function approach with O'Brien-Fleming sequential boundaries to preserve the overall two-sided alpha error rate of

0.05. O'Brien-Fleming alpha levels will be 0.0056 and 0.0482 (2 analyses).If the boundary is crossed at the first interim analysis, the DSMB will convene to decide on termination or continuation of the trial based on efficacy.

10.4 Outcomes

Primary outcome is Executive function. Secondary outcomes include Memory, Attention, Language and MRI measures (perfusion, CVR, WMH, brain connectivity: rs-fMRI and DTI). Additional prespecified outcomes include vascular function measures: CIMT, arterial stiffness, endothelial function and inflammatory markers.

10.5 Data Analysis

The 2 groups will be compared on baseline demographics, education level, physical function (SPPB), IADL, DEX, mood (CESD), cognitive and vascular/inflammatory measures and APO-E genotype. Also, baseline WMH, perfusion, VR, lacunar infarcts, and hippocampal volumes will be compared between the 2 groups using independent t-tests, nonparametric rank-based tests, or chi-square tests as appropriate. We will compare number of AE, SAE and incident dementia in the 2 groups as part of our safety analysis using Fisher's exact test; adverse event categories will utilize MedDRA (Medical Dictionary for Regulatory Activities) coding. To assess possible bias due to selective dropout, baseline comparisons between participants who complete vs. do not complete 12-month outcome assessments will be performed. To assess if equivalent blood pressure was achieved in both groups we will compare mean SBP and DBP in both groups during the follow-up visits.

Outcome Analysis

(a) Candesartan and cognitive/neuroimaging outcomes: General linear models will be used to compare the candesartan and lisinopril groups. [135,136] Our main explanatory variable is the indicator for treatment group. Dependent variables will be the 12-month cognitive and neuroimaging measurements. For the cognitive analyses, our primary outcome is the executive function score (measured by Trail Making test and EXAMINER). An additional measure of executive subdomain will also be derived using item response theory as outlined above in the EXAMINER methodology to account for education, age, and race influences on measures of executive abilities (developed by our collaborator, Dr. Kramer's team).[137,138] For the cognitive outcome Mixed models with repeated measures (3 measurements) will be used. This procedure allows us to model the covariance structure to account for the potential variability across subjects in times of follow-up visits.[135,136] Our main explanatory variable is the indicator for treatment group. The test of treatment effects on trial outcomes will be an interaction of treatment by visit (baseline, 12 months follow-up). The main covariate adjustments will be selected based on baseline statistically significant differences between the 2 randomized groups. The multivariate models of the neuroimaging analysis will include covariates that are significantly differ (at $p<0.05$) between the 2 groups at baseline. We will assess if the effects of randomized treatment are independent of blood pressure by comparing models with and without blood pressure change from baseline to 12 months. Summary statistics will include the model-adjusted mean and 95% confidence interval for each treatment group, and the mean (95% confidence interval) group difference on each 12-month outcome, as well as a p-value for group differences. All tests of significance on treatment group comparisons will use a two-sided alpha of 0.05.

10.6 Sample Size Estimates

Using ANOVA with repeated correlated measures improves power in clinical trials.[139] The 1 year correlation with baseline repeated measures ranged from 0.3 to 0.5 in our pilot data. The effect size for candesartan relative to lisinopril was 0.48 [mean difference 12 (pooled SD 25 sec)] for TMT-B, 0.41 [mean 5.5(13)] for cerebral blood flow and 0.24 [mean 0.30(1.2)] for vasoreactivity to CO_2 in our pilot study. Using a 2-sided $\alpha=0.05$, we estimate that a sample size of 140 will give us 80% power to detect at least 0.24 effect size. To account for dropout rate of 10-15% and a 55% prevalence of eligible subjects, we estimate that we need to screen 332 and enroll 160 to achieve a 140 final sample size.

11. DATA COLLECTION AND QUALITY ASSURANCE

11.1 Data Collection Forms

Data collected during interviews and exams will be documented on trial-specific data forms. Neuroimaging data will be saved in digital formats on a HIPAA-compliant server.

11.2 Data Management

Once a subject is enrolled into the study, he/she will be assigned a unique identifier number and be referred to by initials and the study number only. Only research team members will have access to the files. Data will be entered on a web-based secure trial data system; the data system will include must enter fields, range checks, and simple logic checks. The trial database will include for all variables an electronic data audit of data edits (who, when, and why). A data query report (including missing, out of range, and logic checks) will be generated by the trial statistician weekly; timeliness and completeness of responses to data queries will be monitored. Trial databases will be stored on a secure server. A copy of the master participant list will be kept by the PI in a locked office. The investigators will keep subjects' medical records private as far as the law allows. The IRB and officials of the sponsor/funding agency will have access to these records as needed within legal guidelines. If study results are published in journals or presented at meetings, we will not use the subjects' name. All investigators and research team members have successfully completed the online CITI program for working with Human Subjects in Research.

11.3 Quality Assurance

11.3.1 Training: Research personnel will be trained by the PI and the ADRC investigators and staff. The process of training on data forms completion, neuropsychological assessment and vital sign evaluation will be documented in a training log for each study personnel.

11.3.2 Quality Control: To assess quality of data collected the PI along with another investigator will randomly and on intervals review data obtained. In addition, yearly assessment of personnel competencies in obtaining data (eg Blood pressure checks, neuropsychological assessments etc) will be performed.

11.3.3 Protocol Deviations: Every attempt will be exercised to maintain compliance with the approved study protocol. In the unanticipated event when a deviation is noted, the PI or designated personnel will conduct an investigation about the setting, reasons and potential remedies that need to be instituted to rectify the deviation and prevent future similar instances. The Emory IRB and DSMB will be notified of deviations as set forth in the Emory IRB reporting guidelines.

11.3.4 Monitoring: Monitoring will be provided by the DSMB.

12. PARTICIPANT RIGHTS AND CONFIDENTIALITY

12.1 Institutional Review Board (IRB) Review: This protocol and the informed consent document and any subsequent modifications will be reviewed by the IRB.

12.2 Informed Consent Forms

Subjects will be recruited via various methods including media announcements, screenings, housing facilities visits, and physician offices. By nurturing close relationships with community leaders and health care agencies, and providing free educational programs and health screenings, we expect to be able to recruit the population as described above. All potential subjects will provide informed consent, for screening and inclusion in the study. Any subject who expresses a desire to discontinue participation in the study after providing consent will be removed from the study.

12.3 Cognitive impairment, Capacity Assessment, and Study informants and surrogates

Because of the nature of this research, we may encounter situations where the potential participant, due to his or her cognitive impairment, may not be able to understand the procedures of the study and hence not capable of providing informed consent. Because we are including only executive MCI, we anticipate that the frequency of such a situation is low. During the initial phase, an informal capacity assessment will be administered by the study physician (PI), including the UBACC as described in section 7.2.2. The PI will determine whether the potential participant is able to: "(1) understand the nature of the research and of his or her participation; (2) appreciate the consequences of the participation, including personal consequences; (3) show the ability to consider alternatives, including the option not to participate; and (4) show the ability to make a reasoned choice."[140] If the potential participant lacks the capacity to consent, then assent will be sought from the participant and permission sought from a surrogate.[140] The proxy may be patients' legal guardian (if one exists), next of kin, or familial caregiver according to applicable GA law and consistent with the intent of US 45 C.F.R. 46 and 21 C.F.R. 50 & 56. That person will also need to consent to the study. If either party refuses, we do not enroll the subject. To ensure both the participant and the proxy understand the study protocol, we will ask subjects or legal next of kin to explain in their own words the nature of the study and the procedures involved. Finally, a study informant will also be interviewed. The study informant can be the proxy or a different individual. The criteria for study informant are ability to provide information about the participant, willing to provide information about the participant, and have contact with the participant for at least once a month (in person or telephone).

If there is a clinically significant change in cognitive function that could reasonably change the subject's current status as either capable or not capable, we will reassess the capacity to consent. We will also offer the participant the opportunity to appoint a proxy to make ongoing consent decisions regarding the research project.[140] If capacity is determined to have been lost, we will follow the procedures described in previous paragraph to ensure re-consenting and appointing a proxy.

12.4 Participant Confidentiality

Only the investigators will have access to information about a particular subject. The subject's primary care physician will only be notified if the subject agrees. To maintain confidentiality, subject data will be referenced by number and stored in locked computer files and cabinets. Identifying information about a subject will not be used during the discussion, presentation, or publication of any research data. Only research team members will have access to the files. Data recorded and stored on the computer will be backed up to a disc and stored with the paper files. A copy of the disc will be kept by the Principal Investigator in a locked file drawer. A copy of the master list will be kept by the PI in a locked office. The investigators will keep subjects' medical records private as far as the law allows. The IRB and officials of the sponsor/funding agency will have access to these records as needed within legal guidelines. If study results are published in journals or presented at meetings, we will not use the subjects' name. All investigators and research team members have successfully completed the online CITI program for working with Human Subjects in Research as well as the HIPAA course

12.5 Study Discontinuation

The study may be discontinued at any time by the investigators, the Emory IRB, the NIA, the OHRP, the FDA, or other government agencies as part of their duties to ensure that research participants are protected. A list of reasons for discontinuation of the intervention are provided in section 9. Participants who discontinue the intervention will continue in the study and undergo the scheduled visits.

13. PUBLICATION OF RESEARCH FINDINGS

Publication of the results of this trial will be governed by the policies and procedures of the NIA and Emory.

14. COMMITTEES

Data Safety Monitoring Board (DSMB):

Peter Novak, MD, PhD, DSMB CHAIR
Institution University of Massachusetts Medical School
Department Neurology
University of Massachusetts Medical School
55 Lake Avenue North
Worcester MA 01655
(508) 856-2527
Peter.Novak@umassmemorial.org

Ali Ahmed, MD, MPH
Division of Geriatric Medicine (Internal Medicine)
Washington DC VA Medical Center
50 Irving St NW
Washington, DC 20422, United States
(202)-745-8000
aahmed@uab.edu

Oscar L. Lopez, MD
University of Pittsburgh
Professor of Neurology
(724) 935-1500
LopezOL@upmc.edu

Inmaculada "Chichi" Aban, PhD
Associate Professor
Department of Biostatistics
School of Public Health
The University of Alabama at Birmingham
Office Phone: (205) 934-2732
Fax: (205) 975-2541
caban@uab.edu

15. REFERENCES

1. Vicario A, Martinez CD, Baretto D, Diaz Casale A, Nicolosi L. Hypertension and cognitive decline: impact on executive function. *J Clin Hypertens (Greenwich).* 2005;7(10):598-604.
2. Grigsby J, Kaye K, Shetterly SM, Baxter J, Morgenstern NE, Hamman RF. Prevalence of disorders of executive cognitive functioning among the elderly: findings from the San Luis Valley Health and Aging Study. *Neuroepidemiology.* 2002;21(5):213-220.
3. Royall DR, Espino DV, Polk MJ, Palmer RF, Markides KS. Prevalence and patterns of executive impairment in community dwelling Mexican Americans: results from the Hispanic EPESE Study. *International journal of geriatric psychiatry.* 2004;19(10):926-934.
4. Stuss DT, Levine B. Adult clinical neuropsychology: lessons from studies of the frontal lobes. *Annu Rev Psychol.* 2002;53:401-433.
5. Masterman DL, Cummings JL. Frontal-subcortical circuits: the anatomic basis of executive, social and motivated behaviors. *J Psychopharmacol.* 1997;11(2):107-114.
6. Raz N, Rodrigue KM, Acker JD. Hypertension and the brain: vulnerability of the prefrontal regions and executive functions. *Behav Neurosci.* 2003;117(6):1169-1180.
7. Black SE. Vascular cognitive impairment: epidemiology, subtypes, diagnosis and management. *J R Coll Physicians Edinb.* 2011;41(1):49-56.
8. Bowler JV, Gorelick PB. Advances in vascular cognitive impairment. *Stroke.* 2009;40(5):e315-318.
9. Pantoni L, Gorelick P. Advances in vascular cognitive impairment 2010. *Stroke.* 2011;42(2):291-293.
10. Moorhouse P, Song X, Rockwood K, et al. Executive dysfunction in vascular cognitive impairment in the consortium to investigate vascular impairment of cognition study. *J Neurol Sci.* 2010;288(1-2):142-146.
11. Gottesman RF, Hillis AE. Vascular cognitive impairment. *Behav Neurol.* 2010;22(1-2):1-2.
12. Chiu PY, Liu CH, Tsai CH. Neuropsychiatric manifestations in vascular cognitive impairment patients with and without dementia. *Acta Neurol Taiwan.* 2007;16(2):86-91.
13. Iadecola C, Hachinski V, Rosenberg GA. Vascular cognitive impairment: introduction. *Stroke.* 2010;41(10 Suppl):S127-128.
14. Stephan BC, Matthews FE, Khaw KT, Dufouil C, Brayne C. Beyond mild cognitive impairment: vascular cognitive impairment, no dementia (VCIND). *Alzheimers Res Ther.* 2009;1(1):4.
15. Seo SW, Cho SS, Park A, Chin J, Na DL. Subcortical vascular versus amnestic mild cognitive impairment: comparison of cerebral glucose metabolism. *J Neuroimaging.* 2009;19(3):213-219.
16. Oveisgharan S, Hachinski V. Hypertension, executive dysfunction, and progression to dementia: the canadian study of health and aging. *Archives of neurology.* 2010;67(2):187-192.
17. Rockwood K, Wentzel C, Hachinski V, Hogan DB, MacKnight C, McDowell I. Prevalence and outcomes of vascular cognitive impairment. Vascular Cognitive Impairment Investigators of the Canadian Study of Health and Aging. *Neurology.* 2000;54(2):447-451.
18. Hajjar I, Quach L, Yang F, et al. Hypertension, white matter hyperintensities, and concurrent impairments in mobility, cognition, and mood: the cardiovascular health study. *Circulation.* 2011;123(8):858-865.
19. Wright JW, Harding JW. Brain renin-angiotensin--a new look at an old system. *Prog Neurobiol.* 2011;95(1):49-67.
20. Ciobica A, Bild W, Hritcu L, Haulica I. Brain renin-angiotensin system in cognitive function: pre-clinical findings and implications for prevention and treatment of dementia. *Acta Neurol Belg.* 2009;109(3):171-180.
21. McKinley MJ, Albiston AL, Allen AM, et al. The brain renin-angiotensin system: location and physiological roles. *Int J Biochem Cell Biol.* 2003;35(6):901-918.
22. Wright JW, Reichert JR, Davis CJ, Harding JW. Neural plasticity and the brain renin-angiotensin system. *Neurosci Biobehav Rev.* 2002;26(5):529-552.

23. Culman J, Baulmann J, Blume A, Unger T. The renin-angiotensin system in the brain: an update. *J Renin Angiotensin Aldosterone Syst.* 2001;2(2):96-102.
24. Iwai M, Liu HW, Chen R, et al. Possible inhibition of focal cerebral ischemia by angiotensin II type 2 receptor stimulation. *Circulation.* 2004;110(7):843-848.
25. Reinecke K, Lucius R, Reinecke A, Rickert U, Herdegen T, Unger T. Angiotensin II accelerates functional recovery in the rat sciatic nerve in vivo: role of the AT2 receptor and the transcription factor NF-kappaB. *Faseb J.* 2003;17(14):2094-2096.
26. Horiuchi M, Mogi M. Roles of Activation of Angiotensin II Receptor Subtypes in Ischemic Brain Damage and Cognitive Function. *Br J Pharmacol.* 2010.
27. Horiuchi M, Mogi M, Iwai M. The angiotensin II type 2 receptor in the brain. *J Renin Angiotensin Aldosterone Syst.* 2010;11(1):1-6.
28. Rompe F, Artuc M, Hallberg A, et al. Direct angiotensin II type 2 receptor stimulation acts anti-inflammatory through epoxyeicosatrienoic acid and inhibition of nuclear factor kappaB. *Hypertension.* 2010;55(4):924-931.
29. Wilms H, Rosenstiel P, Unger T, Deuschl G, Lucius R. Neuroprotection with angiotensin receptor antagonists: a review of the evidence and potential mechanisms. *American journal of cardiovascular drugs : drugs, devices, and other interventions.* 2005;5(4):245-253.
30. Steckelings UM, Kaschina E, Unger T. The AT2 receptor--a matter of love and hate. *Peptides.* 2005;26(8):1401-1409.
31. Jugdutt BI, Menon V. Upregulation of angiotensin II type 2 receptor and limitation of myocardial stunning by angiotensin II type 1 receptor blockers during reperfused myocardial infarction in the rat. *J Cardiovasc Pharmacol Ther.* 2003;8(3):217-226.
32. Kobayashi T, Kawamata T, Shibata N, Okada Y, Kobayashi M, Hori T. Angiotensin II type 1 receptor blocker telmisartan reduces cerebral infarct volume and peri-infarct cytosolic phospholipase A(2) level in experimental stroke. *J Neurotrauma.* 2009;26(12):2355-2364.
33. Nagata R, Kawabe K, Ikeda K. Olmesartan, an angiotensin II receptor blocker, restores cerebral hypoperfusion in elderly patients with hypertension. *J Stroke Cerebrovasc Dis.* 2010;19(3):236-240.
34. Oyama N, Yagita Y, Sasaki T, et al. An angiotensin II type 1 receptor blocker can preserve endothelial function and attenuate brain ischemic damage in spontaneously hypertensive rats. *J Neurosci Res.* 2010;88(13):2889-2898.
35. Zazulia AR. Regulation of cerebral blood flow in untreated mild-to-moderate hypertension. *Am J Hypertens.* 2009;22(4):344.
36. Tamaki K, Nakai M, Yokota T, Ogata J. Effects of aging and chronic hypertension on cerebral blood flow and cerebrovascular CO2 reactivity in the rat. *Gerontology.* 1995;41(1):11-17.
37. Girouard H, Iadecola C. Neurovascular coupling in the normal brain and in hypertension, stroke, and Alzheimer disease. *J Appl Physiol.* 2006;100(1):328-335.
38. Hajjar I, Zhao P, Alsop D, Novak V. Hypertension and cerebral vasoreactivity: a continuous arterial spin labeling magnetic resonance imaging study. *Hypertension.* 2010;56(5):859-864.
39. Cohen RA. Hypertension and cerebral blood flow: implications for the development of vascular cognitive impairment in the elderly. *Stroke.* 2007;38(6):1715-1717.
40. Chao LL, Pa J, Duarte A, et al. Patterns of cerebral hypoperfusion in amnestic and dysexecutive MCI. *Alzheimer Dis Assoc Disord.* 2009;23(3):245-252.
41. Silvestrini M, Pasqualetti P, Baruffaldi R, et al. Cerebrovascular reactivity and cognitive decline in patients with Alzheimer disease. *Stroke.* 2006;37(4):1010-1015.
42. Vicenzini E, Ricciardi MC, Altieri M, et al. Cerebrovascular reactivity in degenerative and vascular dementia: a transcranial Doppler study. *European neurology.* 2007;58(2):84-89.
43. Kazama K, Wang G, Frys K, Anrather J, Iadecola C. Angiotensin II attenuates functional hyperemia in the mouse somatosensory cortex. *Am J Physiol Heart Circ Physiol.* 2003;285(5):H1890-1899.
44. Kazama K, Anrather J, Zhou P, et al. Angiotensin II impairs neurovascular coupling in neocortex through NADPH oxidase-derived radicals. *Circ Res.* 2004;95(10):1019-1026.

45. Hajjar I, Sorond F, Hsu YH, Galica A, Cupples LA, Lipsitz LA. Renin angiotensin system gene polymorphisms and cerebral blood flow regulation: the MOBILIZE Boston study. *Stroke.* 2010;41(4):635-640.
46. Taylor WD, Steffens DC, Ashley-Koch A, et al. Angiotensin receptor gene polymorphisms and 2-year change in hyperintense lesion volume in men. *Mol Psychiatry.* 2010;15(8):816-822.
47. van Rijn MJ, Bos MJ, Isaacs A, et al. Polymorphisms of the renin-angiotensin system are associated with blood pressure, atherosclerosis and cerebral white matter pathology. *J Neurol Neurosurg Psychiatry.* 2007;78(10):1083-1087.
48. Iwai M, Inaba S, Tomono Y, et al. Attenuation of focal brain ischemia by telmisartan, an angiotensin II type 1 receptor blocker, in atherosclerotic apolipoprotein E-deficient mice. *Hypertens Res.* 2008;31(1):161-168.
49. Omura-Matsuoka E, Yagita Y, Sasaki T, et al. Postischemic administration of angiotensin II type 1 receptor blocker reduces cerebral infarction size in hypertensive rats. *Hypertens Res.* 2009;32(7):548-553.
50. Wang LY, Larson EB, Sonnen JA, et al. Blood pressure and brain injury in older adults: findings from a community-based autopsy study. *J Am Geriatr Soc.* 2009;57(11):1975-1981.
51. Scuteri A, Nilsson PM, Tzourio C, Redon J, Laurent S. Microvascular brain damage with aging and hypertension: pathophysiological consideration and clinical implications. *J Hypertens.* 2011.
52. Englund E. Neuropathology of white matter lesions in vascular cognitive impairment. *Cerebrovasc Dis.* 2002;13 Suppl 2:11-15.
53. Kramer JH, Reed BR, Mungas D, Weiner MW, Chui HC. Executive dysfunction in subcortical ischaemic vascular disease. *J Neurol Neurosurg Psychiatry.* 2002;72(2):217-220.
54. Gons RA, de Laat KF, van Norden AG, et al. Hypertension and cerebral diffusion tensor imaging in small vessel disease. *Stroke.* 2010;41(12):2801-2806.
55. Nitkunan A, Charlton RA, McIntyre DJ, Barrick TR, Howe FA, Markus HS. Diffusion tensor imaging and MR spectroscopy in hypertension and presumed cerebral small vessel disease. *Magn Reson Med.* 2008;59(3):528-534.
56. Zhou Y, Lin FC, Zhu J, et al. Whole brain diffusion tensor imaging histogram analysis in vascular cognitive impairment. *J Neurol Sci.* 2008;268(1-2):60-64.
57. Xu Q, Zhou Y, Li YS, et al. Diffusion tensor imaging changes correlate with cognition better than conventional MRI findings in patients with subcortical ischemic vascular disease. *Dement Geriatr Cogn Disord.* 2010;30(4):317-326.
58. Burgmans S, van Boxtel MP, Gronenschild EH, et al. Multiple indicators of age-related differences in cerebral white matter and the modifying effects of hypertension. *Neuroimage.* 2010;49(3):2083-2093.
59. Nitkunan A, Barrick TR, Charlton RA, Clark CA, Markus HS. Multimodal MRI in cerebral small vessel disease: its relationship with cognition and sensitivity to change over time. *Stroke.* 2008;39(7):1999-2005.
60. O'Sullivan M. Imaging small vessel disease: lesion topography, networks, and cognitive deficits investigated with MRI. *Stroke.* 2010;41(10 Suppl):S154-158.
61. Tartaglia MC, Zhang Y, Racine C, et al. Executive dysfunction in frontotemporal dementia is related to abnormalities in frontal white matter tracts. *J Neurol.* 2011.
62. O'Sullivan M, Barrick TR, Morris RG, Clark CA, Markus HS. Damage within a network of white matter regions underlies executive dysfunction in CADASIL. *Neurology.* 2005;65(10):1584-1590.
63. Gallinat S, Yu M, Dorst A, Unger T, Herdegen T. Sciatic nerve transection evokes lasting up-regulation of angiotensin AT2 and AT1 receptor mRNA in adult rat dorsal root ganglia and sciatic nerves. *Brain Res Mol Brain Res.* 1998;57(1):111-122.
64. Shenoy UV, Richards EM, Huang XC, Sumners C. Angiotensin II type 2 receptor-mediated apoptosis of cultured neurons from newborn rat brain. *Endocrinology.* 1999;140(1):500-509.

65. Kearney-Schwartz A, Rossignol P, Bracard S, et al. Vascular structure and function is correlated to cognitive performance and white matter hyperintensities in older hypertensive patients with subjective memory complaints. *Stroke.* 2009;40(4):1229-1236.
66. Poels MM, van Oijen M, Mattace-Raso FU, et al. Arterial stiffness, cognitive decline, and risk of dementia: the Rotterdam study. *Stroke.* 2007;38(3):888-892.
67. Scuteri A, Tesauro M, Appolloni S, Preziosi F, Brancati AM, Volpe M. Arterial stiffness as an independent predictor of longitudinal changes in cognitive function in the older individual. *J Hypertens.* 2007;25(5):1035-1040.
68. Triantafyllidi H, Arvaniti C, Lekakis J, et al. Cognitive impairment is related to increased arterial stiffness and microvascular damage in patients with never-treated essential hypertension. *Am J Hypertens.* 2009;22(5):525-530.
69. Wendell CR, Zonderman AB, Metter EJ, Najjar SS, Waldstein SR. Carotid intimal medial thickness predicts cognitive decline among adults without clinical vascular disease. *Stroke.* 2009;40(10):3180-3185.
70. Haley AP, Sweet LH, Gunstad J, et al. Verbal working memory and atherosclerosis in patients with cardiovascular disease: an fMRI study. *J Neuroimaging.* 2007;17(3):227-233.
71. Inoue K, Matsumoto M, Shono T, Toyokawa S, Moriki A. Increased intima media thickness and atherosclerotic plaques in the carotid artery as risk factors for silent brain infarcts. *J Stroke Cerebrovasc Dis.* 2007;16(1):14-20.
72. Shrestha I, Takahashi T, Nomura E, et al. Association between central systolic blood pressure, white matter lesions in cerebral MRI and carotid atherosclerosis. *Hypertens Res.* 2009;32(10):869-874.
73. Sojkova J, Najjar SS, Beason-Held LL, et al. Intima-media thickness and regional cerebral blood flow in older adults. *Stroke.* 2010;41(2):273-279.
74. Tropeano AI, Saleh N, Hawajri N, Macquin-Mavier I, Maison P. Do all antihypertensive drugs improve carotid intima-media thickness? A network meta-analysis of randomized controlled trials. *Fundam Clin Pharmacol.* 2011;25(3):395-404.
75. Wang JG, Staessen JA, Li Y, et al. Carotid intima-media thickness and antihypertensive treatment: a meta-analysis of randomized controlled trials. *Stroke.* 2006;37(7):1933-1940.
76. Zanchetti A, Bond MG, Hennig M, et al. Absolute and relative changes in carotid intima-media thickness and atherosclerotic plaques during long-term antihypertensive treatment: further results of the European Lacidipine Study on Atherosclerosis (ELSA). *J Hypertens.* 2004;22(6):1201-1212.
77. Kunieda T, Minamino T, Nishi J, et al. Angiotensin II induces premature senescence of vascular smooth muscle cells and accelerates the development of atherosclerosis via a p21-dependent pathway. *Circulation.* 2006;114(9):953-960.
78. Mazzolai L, Hayoz D. The renin-angiotensin system and atherosclerosis. *Curr Hypertens Rep.* 2006;8(1):47-53.
79. Vyssoulis GP, Karpanou EA, Kyvelou SM, et al. Beneficial effect of angiotensin II type 1 receptor blocker antihypertensive treatment on arterial stiffness: the role of smoking. *J Clin Hypertens (Greenwich).* 2008;10(3):201-207.
80. Agarwal R. Antihypertensive agents and arterial stiffness: relevance to reducing cardiovascular risk in the chronic kidney disease patient. *Curr Opin Nephrol Hypertens.* 2007;16(5):409-415.
81. Safar ME, Smulyan H. Atherosclerosis, arterial stiffness and antihypertensive drug therapy. *Adv Cardiol.* 2007;44:331-351.
82. Asmar R. Effect of antihypertensive agents on arterial stiffness as evaluated by pulse wave velocity: clinical implications. *American journal of cardiovascular drugs : drugs, devices, and other interventions.* 2001;1(5):387-397.
83. Sun Y. The renin-angiotensin-aldosterone system and vascular remodeling. *Congest Heart Fail.* 2002;8(1):11-16.

84. Ando H, Zhou J, Macova M, Imboden H, Saavedra JM. Angiotensin II AT1 receptor blockade reverses pathological hypertrophy and inflammation in brain microvessels of spontaneously hypertensive rats. *Stroke.* 2004;35(7):1726-1731.
85. Schieffer B, Bunte C, Witte J, et al. Comparative effects of AT1-antagonism and angiotensin-converting enzyme inhibition on markers of inflammation and platelet aggregation in patients with coronary artery disease. *J Am Coll Cardiol.* 2004;44(2):362-368.
86. Ghiadoni L, Virdis A, Magagna A, Taddei S, Salvetti A. Effect of the angiotensin II type 1 receptor blocker candesartan on endothelial function in patients with essential hypertension. *Hypertension.* 2000;35(1 Pt 2):501-506.
87. Kals J, Kampus P, Kals M, et al. Inflammation and oxidative stress are associated differently with endothelial function and arterial stiffness in healthy subjects and in patients with atherosclerosis. *Scand J Clin Lab Invest.* 2008;68(7):594-601.
88. Sata M. Role of chronic inflammation in the pathogenesis of atherosclerosis. *Nihon Yakurigaku Zasshi.* 2011;138(5):182-186.
89. Thorin-Trescases N, Voghel G, Gendron ME, et al. Pathological aging of the vascular endothelium: are endothelial progenitor cells the sentinels of the cardiovascular system? *Can J Cardiol.* 2005;21(12):1019-1024.
90. Boos CJ, Goon PK, Lip GY. Endothelial progenitor cells in the vascular pathophysiology of hypertension: arterial stiffness, ageing and more. *J Hum Hypertens.* 2006;20(7):475-477.
91. Hill JM, Zalos G, Halcox JP, et al. Circulating endothelial progenitor cells, vascular function, and cardiovascular risk. *The New England journal of medicine.* 2003;348(7):593-600.
92. Kong XD, Zhang Y, Liu L, Sun N, Zhang MY, Zhang JN. Endothelial progenitor cells with Alzheimer's disease. *Chin Med J (Engl).* 2011;124(6):901-906.
93. Yao EH, Fukuda N, Matsumoto T, et al. Losartan improves the impaired function of endothelial progenitor cells in hypertension via an antioxidant effect. *Hypertens Res.* 2007;30(11):1119-1128.
94. Fournier A, Achard JM, Boutitie F, et al. Is the angiotensin II Type 2 receptor cerebroprotective? *Curr Hypertens Rep.* 2004;6(3):182-189.
95. Magy L, Vincent F, Faure S, et al. The renin-angiotensin systems: evolving pharmacological perspectives for cerebroprotection. *Curr Pharm Des.* 2005;11(25):3275-3291.
96. Dong Y, Sharma VK, Chan BP, et al. The Montreal Cognitive Assessment (MoCA) is superior to the Mini-Mental State Examination (MMSE) for the detection of vascular cognitive impairment after acute stroke. *J Neurol Sci.* 2010;299(1-2):15-18.
97. Nasreddine ZS, Phillips NA, Bedirian V, et al. The Montreal Cognitive Assessment, MoCA: a brief screening tool for mild cognitive impairment. *J Am Geriatr Soc.* 2005;53(4):695-699.
98. Hachinski V, Iadecola C, Petersen RC, et al. National Institute of Neurological Disorders and Stroke-Canadian Stroke Network vascular cognitive impairment harmonization standards. *Stroke.* 2006;37(9):2220-2241.
99. Johnson JK, Pa J, Boxer AL, Kramer JH, Freeman K, Yaffe K. Baseline predictors of clinical progression among patients with dysexecutive mild cognitive impairment. *Dement Geriatr Cogn Disord.* 2010;30(4):344-351.
100. Pa J, Boxer A, Chao LL, et al. Clinical-neuroimaging characteristics of dysexecutive mild cognitive impairment. *Ann Neurol.* 2009;65(4):414-423.
101. Psaty BM, Lee M, Savage PJ, Rutan GH, German PS, Lyles M. Assessing the use of medications in the elderly: methods and initial experience in the Cardiovascular Health Study. The Cardiovascular Health Study Collaborative Research Group. *Journal of clinical epidemiology.* 1992;45(6):683-692.
102. Lawton MP, Brody EM. Assessment of older people: self-maintaining and instrumental activities of daily living. *The Gerontologist.* 1969;9(3):179-186.
103. Kamei S, Hara M, Serizawa K, et al. Executive dysfunction using behavioral assessment of the dysexecutive syndrome in Parkinson's disease. *Mov Disord.* 2008;23(4):566-573.

104. Vargas ML, Sanz JC, Marin JJ. Behavioral assessment of the dysexecutive syndrome battery (BADS) in schizophrenia: a pilot study in the Spanish population. *Cogn Behav Neurol.* 2009;22(2):95-100.
105. Guralnik JM, Simonsick EM, Ferrucci L, et al. A short physical performance battery assessing lower extremity function: association with self-reported disability and prediction of mortality and nursing home admission. *J Gerontol.* 1994;49(2):M85-94.
106. Schuit AJ, Schouten EG, Westerterp KR, Saris WH. Validity of the Physical Activity Scale for the Elderly (PASE): according to energy expenditure assessed by the doubly labeled water method. *Journal of clinical epidemiology.* 1997;50(5):541-546.
107. Washburn RA, Smith KW, Jette AM, Janney CA. The Physical Activity Scale for the Elderly (PASE): development and evaluation. *Journal of clinical epidemiology.* 1993;46(2):153-162.
108. Pickering TG, Hall JE, Appel LJ, et al. Recommendations for blood pressure measurement in humans and experimental animals: Part 1: blood pressure measurement in humans: a statement for professionals from the Subcommittee of Professional and Public Education of the American Heart Association Council on High Blood Pressure Research. *Hypertension.* 2005;45(1):142-161.
109. Krueger CE, Rosen HJ, Taylor HG, et al. Know thyself: real-world behavioral correlates of self-appraisal accuracy. *Clin Neuropsychol.* 2011;25(5):741-756.
110. Krueger CE, Bird AC, Growdon ME, Jang JY, Miller BL, Kramer JH. Conflict monitoring in early frontotemporal dementia. *Neurology.* 2009;73(5):349-355.
111. Kramer J. Domain Specific Tasks of Executive Function. http://examiner.ucsf.edu/index.htm. USCF. Published ACSESSED 2011. Accessed.
112. Eriksen B, Eriksen C. Effects of noise letters upon the identification of a target letter in a nonsearch task. *Attention, Perception, & Psychophysics.* 1974;16(1):143-149.
113. Lowe C, Rabbitt P. Test/re-test reliability of the CANTAB and ISPOCD neuropsychological batteries: theoretical and practical issues. Cambridge Neuropsychological Test Automated Battery. International Study of Post-Operative Cognitive Dysfunction. *Neuropsychologia.* 1998;36(9):915-923.
114. Owen AM, Downes JJ, Sahakian BJ, Polkey CE, Robbins TW. Planning and spatial working memory following frontal lobe lesions in man. *Neuropsychologia.* 1990;28(10):1021-1034.
115. Ross TP. The reliability of cluster and switch scores for the Controlled Oral Word Association Test. *Arch Clin Neuropsychol.* 2003;18(2):153-164.
116. Kreiner DS, Ryan JJ. Memory and motor skill components of the WAIS-III Digit Symbol-Coding subtest. *Clin Neuropsychol.* 2001;15(1):109-113.
117. Wechsler D. *WMS-R : Wechsler Memory Scale--Revised : manual.* San Antonio: Psychological Corp. : Harcourt Brace Jovanovich; 1987.
118. Mack WJ, Freed DM, Williams BW, Henderson VW. Boston Naming Test: shortened versions for use in Alzheimer's disease. *J Gerontol.* 1992;47(3):P154-158.
119. Canning SJ, Leach L, Stuss D, Ngo L, Black SE. Diagnostic utility of abbreviated fluency measures in Alzheimer disease and vascular dementia. *Neurology.* 2004;62(4):556-562.
120. Radloff LS. The CES-D Scale: A Self-Report Depression Scale for Research in the General Population. *Applied Psychological Measurement.* 1977;1(3):385-401.
121. Benicky J, Sanchez-Lemus E, Pavel J, Saavedra JM. Anti-inflammatory effects of angiotensin receptor blockers in the brain and the periphery. *Cell Mol Neurobiol.* 2009;29(6-7):781-792.
122. Krejcy K, Eichler HG, Jilma B, et al. Influence of angiotensin II on circulating adhesion molecules and blood leukocyte count in vivo. *Can J Physiol Pharmacol.* 1996;74(1):9-14.
123. Nielsen HM, Londos E, Minthon L, Janciauskiene SM. Soluble adhesion molecules and angiotensin-converting enzyme in dementia. *Neurobiol Dis.* 2007;26(1):27-35.
124. Prasad A, Koh KK, Schenke WH, et al. Role of angiotensin II type 1 receptor in the regulation of cellular adhesion molecules in atherosclerosis. *Am Heart J.* 2001;142(2):248-253.
125. Tsutamoto T, Wada A, Maeda K, et al. Angiotensin II type 1 receptor antagonist decreases plasma levels of tumor necrosis factor alpha, interleukin-6 and soluble adhesion molecules in patients with chronic heart failure. *J Am Coll Cardiol.* 2000;35(3):714-721.

126. Marchesi C, Paradis P, Schiffrin EL. Role of the renin-angiotensin system in vascular inflammation. *Trends Pharmacol Sci.* 2008;29(7):367-374.
127. Graninger M, Reiter R, Drucker C, Minar E, Jilma B. Angiotensin Receptor Blockade Decreases Markers of Vascular Inflammation. *Journal of cardiovascular pharmacology.* 2004;44(3):335-339.
128. Trollor JN, Smith E, Agars E, et al. The association between systemic inflammation and cognitive performance in the elderly: the Sydney Memory and Ageing Study. *Age (Dordr).* 2011.
129. Fadini GP, Baesso I, Albiero M, Sartore S, Agostini C, Avogaro A. Technical notes on endothelial progenitor cells: ways to escape from the knowledge plateau. *Atherosclerosis.* 2008;197(2):496-503.
130. Vasa M, Fichtlscherer S, Aicher A, et al. Number and migratory activity of circulating endothelial progenitor cells inversely correlate with risk factors for coronary artery disease. *Circ Res.* 2001;89(1):E1-7.
131. Jeste DV, Palmer BW, Appelbaum PS, et al. A new brief instrument for assessing decisional capacity for clinical research. *Arch Gen Psychiatry.* 2007;64(8):966-974.
132. Appelbaum PS, Grisso T. *MacArthur competence assessment tool for clinical research (MacCAT-CR).* Sarasota, FL: Professional Resource Press; 2001.
133. Stroup S, Appelbaum P, Swartz M, et al. Decision-making capacity for research participation among individuals in the CATIE schizophrenia trial. *Schizophr Res.* 2005;80(1):1-8.
134. Marra C, Ferraccioli M, Vita MG, Quaranta D, Gainotti G. Patterns of cognitive decline and rates of conversion to dementia in patients with degenerative and vascular forms of MCI. *Curr Alzheimer Res.* 2011;8(1):24-31.
135. Diggle P, Liang K, Zeger S. *Analysis of Longitudinal Data.*: Oxford University Press; 1994.
136. McCullagh P, Nelder J. *Generalized Liner Models.* London.: Chapman and Hall.; 1987.
137. Crane PK, Narasimhalu K, Gibbons LE, et al. Composite scores for executive function items: demographic heterogeneity and relationships with quantitative magnetic resonance imaging. *J Int Neuropsychol Soc.* 2008;14(5):746-759.
138. Crane PK, Narasimhalu K, Gibbons LE, et al. Item response theory facilitated cocalibrating cognitive tests and reduced bias in estimated rates of decline. *Journal of clinical epidemiology.* 2008;61(10):1018-1027 e1019.
139. Tran ZV. Estimating Sample Size in Repeated-Measures Analysis of Variance. *Measurement in Physical Education and Exercise Science.* 1997;1(1):89-102.
140. Research consent for cognitively impaired adults: recommendations for institutional review boards and investigators. *Alzheimer Dis Assoc Disord.* 2004;18(3):171-175.

STATISTICAL ANALYSIS PLAN

CANDESARTAN VS. LISINOPRIL EFFECTS ON THE BRAIN (CALIBREX)

Darius McDaniel, MSPH

Neeta Shenvi, MS

John Hanfelt, PhD

Ihab Hajjar, MD

Emory University School of Medicine

Table of Contents

1. BACKGROUND AND RATIONALE (Study protocol) **3**

2. STUDY OBJECTIVES **3**

2.1. Aim 1 3

2.2. Aim 2 3

2.3. Aim 3 3

3. SELECTION AND ENROLLMENT **3**

4. DATA ANALYSIS **3**

3.1. Outcome Analysis 5

5. STATISTICAL METHODOLOGY **5**

5.1. Statistical Procedures 5

6. SENSITIVITY ANALYSES **5**

7. QUALITY CHECK PLANS **6**

8. PROGRAMMING PLANS **6**

9. TABLES AND FIGURES **6**

9.1. Tables 6

9.2. Figures 6

1. BACKGROUND AND RATIONALE (Study protocol)

2. STUDY OBJECTIVES

Our overall aim is to conduct a 1-year double blind randomized control trial comparing candesartan to lisinopril in individuals with hypertension and executive mild cognitive impairment. Our specific aims are:

2.1. Aim 1

Investigate the effect of candesartan compared to lisinopril on the progression of cognitive function evaluated by a battery of neuropsychological tests assessing executive function, memory, attention and cognitive speed in hypertensive older adults with executive mild cognitive impairment.
Hypothesis 1: Candesartan is associated with less decline in executive function compared to lisinopril.

2.2. Aim 2

Investigate the effect of candesartan compared to lisinopril on cerebral perfusion, cerebrovascular reserve (vasoreactivity to carbon dioxide) assessed by arterial spin labeling MRI and microvascular brain injury reflected by diffusion tensor imaging measures of connectivity and white matter hyperintensities or lesions (WML).

Hypothesis 2: Candesartan is associated with less decline in cerebral perfusion, vasoreactivity to carbon dioxide, and evidence of microvascular brain injury (white matter integrity) compared to lisinopril.

2.3. Aim 3

Identify underlying mechanisms explaining the effects of candesartan on neuroimaging (AIM 3A) and cognitive (AIM 3B) outcomes relative to lisinopril including effects on arterial stiff- ness, atherosclerosis (carotid intima media thickness, CIMT), and vascular inflammatory and endothelial markers.

Hypotheses 3: Relative to lisinopril, treatment with candesartan is associated with lower CIMT, arterial stiffness, and inflammatory and endothelial function markers at 12 months, which in turn are associated with improved cerebral perfusion, vasoreactivity, and white matter integrity measures (Hypotheses 3A) as well as less decline in executive function (Hypotheses 3B) at 12 months.

3. SELECTION AND ENROLLMENT

Inclusion Criteria:

(1) Age: 55 years or older;

(2) Hypertension: SBP ≥140 mm Hg or DBP ≥ 90 mm or receiving antihypertensive medications;

(3) Executive MCI will be defined using these criteria;

(a) The Montreal Cognitive Assessment (MoCA) score less than or equal to 26

(b) Executive dysfunction: A performance at the 10th percentile or below on at least one of four screening tests for executive function: Trail Making Test, Part B (TMT-B), modified Stroop interference, Digit Span and Digit Sequencing, and Letter fluency

(c) Minimal Functional limitation as reflected by the Functional Assessment Questionnaire (FAQ) ≤7.

Exclusion Criteria:

(1) Intolerance to candesartan or lisinopril;

(2) SBP >200 or DBP >110 mm Hg;

(3) Renal disease or hyperkalemia (Serum Cr >1.99 mg/dl or K >5.5 meq/dl);

(4) Active medical or psychiatric problems (An active problem is one that requires medical attention and is deemed as a safety risk by the study physician, for example current cancer treatment, infections such as pneumonia, current gastrointestinal or other internal bleeding, HIV treatment, hematological disease, Parkinson's disease, multiple sclerosis);

(5) Uncontrolled congestive heart failure [shortness of breath at rest or evidence of pulmonary edema on exam);

(6) History of stroke in the past 3 years (self-reported clinical stroke within the past 3 years/ incidental infarcts on MRI scanning is allowed);

(7) Inability to perform the study procedures (those with contraindications for MRI will be enrolled but will not undergo the MRI; those with history of asthma, COPD, or significant pulmonary disease will not undergo the CO_2-reactivity protocol);

(8) Women of childbearing potential;

(9) A diagnosis of dementia self-report or care-giver report;

(10) In those who lack decision capacity, a study surrogate who can sign on their behalf will be required. Since we are enrolling only those with MCI, we anticipate that most participants will have decision capacity;

(11) Current use of Lithium, as most antihypertensive classes may lead to increased lithium toxic levels.

4. DATA ANALYSIS

Primary outcome is executive function (TMT and EXAMINER scores). Secondary outcomes

include memory, attention, language and MRI measures (perfusion, CVR, WMH, brain connectivity: rs-fMRI and DTI). Additional prespecified outcomes include vascular function measures: CIMT, arterial stiffness, endothelial function and inflammatory markers.

The 2 groups will be compared on baseline demographics, education level, physical function (SPPB), IADL, DEX, mood (CESD), cognitive and vascular/inflammatory measures and APO-E genotype. Also, baseline WMH, perfusion, VR, lacunar infarcts and hippocampal volumes will be compared between the 2 groups using independent t-tests, nonparametric rank-based tests, or chi-square tests as appropriate. We will compare number of AE, SAE and incident dementia in the 2 groups as part of our safety analysis using Fisher's exact test; adverse event categories will utilize MedDRA (Medical Dictionary for Regulatory Activities) coding. To assess possible bias due to selective dropout, baseline comparisons between participants who complete vs. do not complete 12-month outcome assessments will be performed. To assess if equivalent blood pressure was achieved in both groups, we will compare mean SBP and DBP in both groups during the follow-up visits.

4.1. Outcome Analysis

Mixed models with repeated measures (MMRM) will be used to compare the outcomes between candesartan and lisinopril groups. Our main explanatory variable is the indicator for treatment group. Dependent variables will be the selected outcomes measured 3 times over the 12 months period. For the cognitive analyses, our primary outcome is executive function (measured by Trail Making Test, parts A, B and B-A and EXAMINER). The test of treatment effects on trial outcomes will be an interaction of treatment by visit (baseline, 6- and 12-months follow-up). The main covariate adjustments will be selected based on baseline statistically significant differences between the 2 randomized groups as well as stratification variables. We will assess if the effects of randomized treatment are independent of blood pressure by comparing models with and without blood pressure change from baseline to 12 months. Summary statistics will include the model-adjusted mean (LSM) and 95% confidence interval for each treatment group at each visit, and the mean (95% confidence interval) group difference on each outcome over the 12 months (effect size). All tests of significance on treatment group comparisons will use a two-sided alpha of .05.

5. STATISTICAL METHODOLOGY

5.1. Statistical Procedures

Chi-square test and Student's t-test will be performed on categorical and continuous data respectively. For primary outcome analysis, mixed models will be used to evaluate changes. The model will be adjusted for trial group assignment and the interaction between time (visits) and trial group assignment. Least square means between trial groups by visit will also be estimated from this model. An unstructured covariance matrix will be used to model the correlation for repeated measurements.

6. SENSITIVITY ANALYSES

For outcome measures that differed at baseline between the 2 groups, a sensitivity analysis will be conducted by adjusting the baseline measure for that outcome. For those instances, we will provide unadjusted and adjusted results.

7. QUALITY CHECK PLANS

To assess quality of data collected, the PI along with another investigator will randomly and on intervals review data obtained. In addition, yearly assessment of personnel competencies in obtaining data (e.g. Blood pressure checks, neuropsychological assessments, etc.) will be performed. Data entered into redcap will be checked by a second operator to match the source documents with the database.

8. PROGRAMMING PLANS

SAS 9.4 will be used for all analyses by study statisticians.

9. TABLES AND FIGURES

9.1. Tables

(1) Demographics Characteristics
(2) Adverse Events
(3) Complete vs. Dropout
(4) Outcomes Linear mixed method results

9.2. Figures

(1) Consort Diagram
(2) Potassium, Creatinine and Blood pressure
(3) Cognitive outcomes
(4) Imaging outcomes
