# Supplementary material for: Effects of Candesartan vs Lisinopril on Neurocognitive Function in Older Adults With Executive Mild Cognitive Impairment: A Randomized Clinical Trial
Source: JAMA Netw Open. 2020 Aug 6;3(8):e2012252. doi: 10.1001/jamanetworkopen.2020.12252 (PMC7411539; doi:10.1001/jamanetworkopen.2020.12252)
Supplement: Supplement 2. — eTable 1. Clinical, Demographic, and Cognitive Characteristics Comparisons Between Participants Who Completed the Study vs Those Who Dropped Out eTable 2. Participants Using Study and Open-Label Antihypertensive Medications eTable 3. Blood Pressure, Pulse Rate, and Blood Biochemistry During the Follow-Up Period eTable 4. Adverse Events Reported During Trial Period by Treatment Group eTable 5. Primary and Secondary Outcomes and Treatment Effects From the Final Mixed Model With Repeated Measures Models eFigure. Serum Potassium and Creatinine Levels Stratified by Treatment Group [file jamanetwopen-3-e2012252-s002.pdf]

Supplementary Online Content

Hajjar I, Okafor M, McDaniel D, et al. Effects of candesartan vs lisinopril on neurocognitive function in older adults with executive mild cognitive impairment: a randomized clinical trial. *JAMA Netw Open*. 2020;3(8):e2012252. doi:10.1001/jamanetworkopen.2020.12252

eTable 1. Clinical, Demographic, and Cognitive Characteristics Comparisons Between Participants Who Completed the Study vs Those Who Dropped Out

eTable 2. Participants Using Study and Open-Label Antihypertensive Medications

eTable 3. Blood Pressure, Pulse Rate, and Blood Biochemistry During the Follow-Up Period

eTable 4. Adverse Events Reported During Trial Period by Treatment Group

eTable 5. Primary and Secondary Outcomes and Treatment Effects From the Final Mixed Model With Repeated Measures Models

eFigure. Serum Potassium and Creatinine Levels Stratified by Treatment Group

This supplementary material has been provided by the authors to give readers additional information about their work.

© 2020 Hajjar I et al. *JAMA Network Open.*

eTable 1. Clinical, Demographic, and Cognitive Characteristics Comparisons Between Participants Who Completed the Study vs Those Who Dropped Out

	**Lisinopril**			**Candesartan**			***Lisinopril vs Candesartan p-value***
	**Completed (n=64)**	**Dropout (n=25)**	***p-value***	**Complete (n=77)**	**Dropout (n=10)**	***p-value***	
**Demographics**							
Age(years), mean (SD)	66.2 (7.0)	64.7 (7.8)	0.32	65.4 (7.8)	71.5 (10.9)	0.11	0.86
Female:	35(54.7%)	19(76.0%)	0.06	43(55.8%)	4(40.0%)	0.34	0.27
Ethnicity:			0.51			0.53	0.68
Non-Hispanic or Latino	61(95.3%)	22(91.7%)		74(96.1%)	10(100%)		
Hispanic or Latino	3(4.7%)	2(8.3%)		3(3.9%)	0(0.0%)		
Race:			0.21			0.07	0.05
White	24(37.5%)	7(28.0%)		28(36.4%)	1(10.0%)		
Black or African American	40(62.5%)	17(68.0%)		48(62.3%)	8(80.0%)		
Other	0(0.0%)	1(4.0%)		1(1.3%)	1(10.0%)		
Years of education, mean (SD)	15.4 (2.7)	15.4 (2.1)	0.99	14.8 (2.6)	14.4 (2.8)	0.65	0.87
Marital status:			0.15			0.58	0.15
Married	28(44.4%)	5(20.8%)		29(37.7%)	1(12.5%)		
Divorced/Separated	21(33.3%)	11(45.8%)		27(35.1%)	5(62.5%)		
Widowed	10(15.9%)	4(16.7%)		11(14.3%)	1(12.5%)		
Single/Never Married	4(6.3%)	3(12.5%)		9(11.7%)	1(12.5%)		
Other	0(0.0%)	1(4.2%)		1(1.3%)	0(0.0%)		
Subject Living with:			0.31			0.15	0.05
Alone	22(34.4%)	11(44.0%)		28(36.4%)	6(60.0%)		
With spouse or partner	29(45.3%)	6(24.0%)		35(45.5%)	1(10.0%)		
With child or other family	10(15.6%)	6(24.0%)		12(15.6%)	3(30.0%)		
Other	3(4.7%)	2(8.0%)		2(2.6%)	0(0.0%)		
Body mass index, mean (SD)	32.8 (7.9)	32.2 (5.2)	0.75	32.6 (7.0)	31.2 (6.5)	0.69	0.96
Family history of dementia:	12(19.0%)	6(25.0%)	0.54	26(35.6%)	1(10.0%)	0.10	0.38
**Blood Pressure and Pulse Rate Readings, mean (SD)**							
Sitting systolic BP	144.2 (19.1)	148.2 (25.4)	0.56	138.9 (20.9)	150.5 (21.3)	0.08	0.08
Standing systolic BP, at 3min	150.3 (23.2)	152.3 (20.7)	0.25	145.5 (21.4)	156.5 (26.9)	0.16	0.05
Sitting diastolic BP	86.2 (13.6)	82.7 (12.1)	0.40	83.4 (12.9)	86.2 (13.4)	0.50	0.98
Standing diastolic BP, at 3min	92.9 (14.9)	89.7 (11.2)	0.61	90.4 (12.7)	95.1 (23.3)	0.97	0.73
Sitting pulse	71.5 (11.5)	68.7 (12.5)	0.17	68.5 (9.8)	77.7 (14.4)	0.021*	0.77
Standing pulse, at 3min	79.7 (13.2)	76.1 (14.3)	0.19	76.8 (11.2)	88.3 (18.0)	0.017*	0.83
**Cognitive Status, mean (SD)**							
MoCA score	21.7 (3.5)	21.6 (3.5)	0.83	21.4 (3.5)	21.1 (2.7)	0.52	0.71
TMT, A, completion time (seconds)	38.0 (14.0)	38.7 (16.8)	0.91	39.5 (15.6)	49.0 (15.9)	0.048*	0.42
TMT, B, completion time (seconds)	127.9 (78.0)	137.4 (81.5)	0.42	150.3 (84.2)	200.0 (91.9)	0.08	0.30
TMT, B - A (seconds)	89.8 (74.9)	98.7 (72.7)	0.37	110.7 (77.0)	151.0 (91.9)	0.16	0.37
HVLT, immediate recall	22.7 (4.4)	23.0 (4.8)	0.68	21.9 (4.6)	18.8 (4.9)	0.06	0.69
HVLT, delayed recall	6.9 (3.4)	7.2 (2.9)	0.99	6.6 (3.2)	5.8 (2.3)	0.35	0.80
HVLT, recognition discrimination index	11.1 (1.2)	11.2 (1.0)	0.86	10.9 (1.9)	10.2 (1.6)	0.11	0.60
HVLT, retention	74.7 (31.5)	79.3 (27.6)	0.91	71.6 (31.8)	67.7 (22.1)	0.44	0.97
Executive composite	0.1 (0.5)	0.1 (0.4)	0.74	-0.0 (0.6)	-0.2 (0.6)	0.51	0.79

© 2020 Hajjar I et al. *JAMA Network Open.*

		Lisinopril			Candesartan			Lisinopril vs Candesartan p-value
		Completed (n=64)	Dropout (n=25)	*p-value*	Complete (n=77)	Dropout (n=10)	*p-value*	
Boston Naming Test		13.7 (1.4)	13.5 (1.2)	0.26	13.1 (2.1)	13.6 (1.6)	0.48	0.93
Digital Span Test, forward		8.9 (2.0)	8.9 (2.2)	0.85	9.0 (2.1)	8.7 (2.0)	0.75	0.95
Digital Span Test, backward		5.3 (1.9)	4.9 (2.0)	0.23	5.0 (1.9)	4.9 (1.0)	0.96	0.40
CESD score		9.6 (8.0)	11.5 (10.5)	0.46	11.1 (9.1)	12.6 (13.5)	0.97	0.66
PASE score		202.6 (169.7)	168.9 (142.9)	0.26	219.5 (202.3)	141.7 (72.3)	0.63	0.29
Dysexecutive Questionnaire score		12.1 (9.7)	12.6 (8.1)	0.54	14.2 (11.7)	15.3 (10.4)	0.61	0.57
Instrumental Activities of Daily Living score		7.8 (0.8)	7.5 (1.3)	0.80	7.9 (2.1)	7.4 (0.8)	0.10	0.21
Functional Activities Questionnaire score		0.8 (1.1)	0.5 (1.0)	0.37	0.9 (1.6)	0.7 (1.2)	0.77	0.43
MCI Category				0.54			0.97	0.38
Executive		39(60.9%)	17(68.0%)		38(49.4%)	5(50.0%)		
Mixed, Executive and Amnestic		25(39.1%)	8(32.0%)		39(50.6%)	5(50.0%)		
**Blood Chemistry, mean (SD)**								
WBC (1000/uL)		6.5 (2.7)	6.0 (1.9)	0.59	6.2 (1.8)	6.6 (1.8)	0.33	0.94
Hemoglobin (g/dL)		13.4 (1.4)	13.0 (1.3)	0.21	13.4 (1.3)	12.9 (1.0)	0.17	0.06
Platelets (1000/uL)		231.8 (58.3)	247.0 (44.2)	0.26	237.0 (65.1)	241.4 (85.9)	0.76	0.30
Sodium (mmol/L)		140.4 (2.4)	139.9 (2.0)	0.38	140.1 (2.7)	140.2 (3.1)	0.78	0.60
Potassium (mEq/L)		4.1 (0.5)	4.1 (0.6)	0.72	4.1 (0.5)	4.3 (0.6)	0.61	0.95
Creatinine (mg/dL)		0.9 (0.2)	1.0 (0.3)	0.57	0.9 (0.2)	1.1 (0.3)	0.12	0.29
**Clinical Diagnosis**								
Diabetes mellitus		21(33.3%)	6(24.0%)	0.39	22(28.6%)	2(20.0%)	0.57	0.36
Heart disease, coronary and valvular		21(32.8%)	8(32.0%)	0.94	18(23.4%)	0(0.0%)	0.09	0.57
Hyperlipidemia:		34(54.0%)	12(50.0%)	0.74	45(59.2%)	9(90.0%)	0.06	0.60
Remote Stroke:		5(7.8%)	1(4.0%)	0.52	5(6.5%)	0(0.0%)	0.41	0.35
Depression‡		17(26.6%)	6(25.0%)	0.88	19(24.7%)	3(30.0%)	0.72	0.91
**Pre-Randomization Medications**								
ACEI		26(40.6%)	6(24.0%)	0.14	28(36.4%)	5(50.0%)	0.40	0.45
ARB		14(21.9%)	6(24.0%)	0.83	15(19.5%)	2(20.0%)	0.97	0.77
Either ACEI or ARB:		39(60.9%)	12(48.0%)	0.27	43(55.8%)	7(70.0%)	0.39	0.68
Cholinesterase inhibitors or Memantine:		3(4.7%)	0(0.0%)	0.27	1(1.3%)	0(0.0%)	0.72	0.31
Any antihypertensive medication:		52(81.3%)	20(80.0%)	0.89	70(90.9%)	9(90.0%)	0.93	0.58
Anti-diabetic:		19(29.7%)	5(20.0%)	0.35	21(27.3%)	2(20.0%)	0.62	0.32
Lipid Lowering agent:		27(42.2%)	9(36.0%)	0.59	42(54.5%)	6(60.0%)	0.74	0.52
Anti-depression:		16(25.0%)	5(20.0%)	0.62	17(22.1%)	2(20.0%)	0.88	0.67

Abbreviations: ACEI: angiotensin converting enzyme inhibitors; ARB: angiotensin II receptor blockers; BP: blood pressure; CESD: Center for Epidemiologic Studies Depression scale; EXAMINER: Executive Abilities: Methods and Instruments for Neurobehavioral Evaluation and Research; HVLT-R: Hopkins Verbal Learning Test - Revised; MCI: mild cognitive impairment; MoCA: Montreal Cognitive Assessment; PASE: Physical Activity Scale for the Elderly; SD: standard deviation; TMT: Trail Making Test; WBC: white blood cells.
All values reported are frequency (percentage) or mean (SD).

© 2020 Hajjar I et al. *JAMA Network Open.*

eTable 2. Participants Using Study and Open-Label Antihypertensive Medications

Study Medication Step and Dose	Overall (N=176) No. (%)	Candesartan (n=87) No. (%)	Lisinopril (n=89) No. (%)
Candesartan 8 mg / Lisinopril 10 mg	86 (48.9)	42 (48.3)	44 (49.4)
Candesartan 16 mg / Lisinopril 20 mg	30 (17.0)	15 (17.2)	15 (16.9)
Candesartan 32 mg / Lisinopril 40 mg	14 (8.0)	7 (8.0)	7 (7.9)
Study intervention + HCTZ 12.5 mg	27 (15.3)	13 (14.9)	14 (15.7)
Study intervention + HCTZ 25 mg	7 (4.0)	5 (5.7)	2 (2.2)
Study intervention + HCTZ 25 mg + Amlodipine 2.5 mg	9 (5.1)	4 (4.6)	5 (5.6)
Study intervention + HCTZ 25 mg + Amlodipine 5 mg	2 (1.1)	0 (0.0)	2 (2.2)
Study intervention + HCTZ 25 mg + Amlodipine10 mg	1 (0.6)	1 (1.1)	0 (0.0)

Abbreviations: HCTZ: hydrochlorothiazide.

© 2020 Hajjar I et al. *JAMA Network Open.*

eTable 3. Blood Pressure, Pulse Rate, and Blood Biochemistry During the Follow-Up Period

**Outcome**	**Visit**	**Lisinopril (n=89) Mean (SD)**	**Candesartan (n=87) Mean (SD)**	***P-value for treatment effect***
Sitting systolic BP (mmHg), mean	Baseline	145.33 (20.98)	140.25 (21.12)	*.05*
	3 Month	130.93 (17.32)	131.6 (16.34)	
	6 Month	129.17 (13.72)	128.41 (16.18)	
	12 Month	129.95 (17.38)	134.31 (19.86)	
Standing systolic BP (mmHg), at 1 min	Baseline	152.19 (22.42)	146.66 (20.6)	*.21*
	3 Month	134.58 (20.23)	134.04 (16.34)	
	6 Month	135.67 (16.27)	134.11 (16.53)	
	12 Month	138.52 (18.89)	139.25 (19.62)	
Standing systolic BP (mmHg), at 3 min	Baseline	150.89 (22.42)	146.72 (22.21)	*.27*
	3 Month	138.35 (20.94)	137.3 (17.99)	
	6 Month	136.64 (17.99)	137.16 (19.47)	
	12 Month	137.28 (19.31)	140.45 (19.54)	
Sitting diastolic BP (mmHg), mean	Baseline	85.23 (13.22)	83.75 (12.89)	*.39*
	3 Month	78.87 (12.34)	78.86 (11.27)	
	6 Month	77.09 (10.36)	75.92 (9.26)	
	12 Month	77.02 (10.85)	78.32 (12.4)	
Standing diastolic BP (mmHg), at 1 min	Baseline	91.23 (14.08)	90.78 (13.66)	*.87*
	3 Month	83.71 (13.08)	84.03 (11.52)	
	6 Month	83.92 (11.04)	82.92 (11.1)	
	12 Month	84.85 (10.73)	85.21 (13.12)	
Standing diastolic BP (mmHg), at 3 min	Baseline	91.96 (13.98)	90.92 (14.23)	*.35*
	3 Month	84.67 (13.92)	85.14 (12.42)	
	6 Month	84.09 (10.9)	83.25 (12.68)	
	12 Month	84.26 (12.49)	86.53 (14.05)	
Sitting pulse (beats/min), mean	Baseline	70.72 (11.79)	69.58 (10.74)	*.75*
	3 Month	72.68 (12.53)	70.73 (10.07)	
	6 Month	73.15 (12.63)	70.66 (10.78)	
	12 Month	70.23 (12.2)	69.23 (10.61)	
Standing pulse (beats/min), at 1 min	Baseline	77.49 (13.34)	77.71 (13.26)	*.98*
	3 Month	80.32 (13.33)	78.93 (12.07)	

© 2020 Hajjar I et al. *JAMA Network Open.*

	6 Month	80.2 (14.78)	78.99 (12.37)	
	12 Month	78.54 (15.14)	77.05 (12.28)	
Standing pulse (beats/min), at 3 min	Baseline	78.65 (13.55)	78.13 (12.58)	*.94*
	3 Month	79.89 (13.12)	78.11 (11.51)	
	6 Month	80.77 (13.97)	79.16 (12.45)	
	12 Month	78.69 (14.5)	76.15 (11.23)	
Serum creatinine (mg/dL)	Baseline	0.93 (0.25)	0.94 (0.2)	*.58*
	3 Month	0.96 (0.25)	0.98 (0.23)	
	6 Month	0.97 (0.26)	0.98 (0.22)	
	12 Month	0.97 (0.27)	0.96 (0.21)	
Serum potassium (mmol/L)	Baseline	4.14 (0.55)	4.14 (0.55)	*.45*
	3 Month	4.22 (0.5)	4.35 (0.51)	
	6 Month	4.38 (0.72)	4.31 (0.49)	
	12 Month	4.25 (0.47)	4.27 (0.48)	

Abbreviation: BP: blood pressure, SD: standard deviation

© 2020 Hajjar I et al. *JAMA Network Open.*

eTable 4. Adverse Events Reported During Trial Period by Treatment Group

**Symptom**	**Lisinopril n (%)**	**Candesartan n (%)**	***P-value***
Cough	24 (27%)	7 (8%)	*.005*
Headache	22 (25%)	18 (21%)	*.92*
Dizziness or lightheadedness	14 (16%)	15 (17%)	*.49*
Edema/swelling	12 (13%)	13 (15%)	*.50*
General Pain	9 (10%)	7 (8%)	*.87*
Excessive tiredness, asthenia, fatigue, weakness	7 (8%)	9 (10%)	*.38*
Nasal/sinus drainage	6 (7%)	1 (1%)	*.09*
Palpitation	4 (4%)	4 (5%)	*.80*
Urinary frequency	3 (3%)	4 (5%)	*.54*
Leg Cramps	2 (2%)	1 (1%)	*.67*
Insomnia	2 (2%)	0 (0%)	*.19*
Runny nose	2 (2%)	2 (2%)	*.86*
Unusual bleeding or bruising	1 (1%)	0 (0%)	*.36*
Falling	1 (1%)	3 (3%)	*.24*
Dry mouth	1 (1%)	1 (1%)	*.90*
Flatulence, gas or bloating	1 (1%)	0 (0%)	*.36*
Loss of appetite	1 (1%)	0 (0%)	*.36*
Nausea	1 (1%)	2 (2%)	*.46*
Shortness of breath	1 (1%)	0 (0%)	*.36*
Wheezing	1 (1%)	0 (0%)	*.36*
Severe elevation in systolic BP >200 mmHg	1 (1%)	0 (0%)	*.36*
Rash	1 (1%)	8 (9%)	*.008*
Hoarseness	1 (1%)	1 (1%)	*.90*
Sore throat	1 (1%)	1 (1%)	*.90*
Sweating	1 (1%)	0 (0%)	*.36*
Fainting	0 (0%)	1 (1%)	*.28*
Worsening renal function (serum creatinine >2.5)	0 (0%)	1 (1%)	*.28*
Low potassium (<3.0)	0 (0%)	2 (2%)	*.12*

© 2020 Hajjar I et al. *JAMA Network Open.*

eTable 5. Primary and Secondary Outcomes and Treatment Effects From the Final Mixed Model With Repeated Measures Models

Outcome	Visit	Lisinopril (n=89) LS Mean (SE)[a]	Candesartan (n=87) LS Mean (SE)[a]	***Treatment Effect (95% CI)[b]***
Primary Outcome (Executive measures)				
TMT Part A (seconds)	Baseline	37.40 (1.81)	40.11 (1.84)	*1.19 (-1.44, 3.83)*
	6 Month	39.94 (2.48)	42.07 (2.39)	
	12 Month	37.08 (2.31)	41.92 (2.22)	
TMT Part B (seconds)	Baseline	139.7 (5.18)	143.11 (5.21)	*-12.81 (-22.55, -3.06)*
	6 Month	160.03 (5.8)	139.78 (5.48)	
	12 Month	150.15 (5.87)	128.28 (5.44)	
TMT Part B - A (seconds)	Baseline	99.79 (5.19)	103.94 (5.22)	*-14.32 (-24.16, -4.49)*
	6 Month	118.38 (5.83)	98.97 (5.5)	
	12 Month	111.37 (5.89)	87.23 (5.46)	
EXAMINER executive composite	Baseline	0.12 (0.06)	-0.01 (0.06)	*-0.03 (-0.08, 0.03)*
	6 Month	0.18 (0.06)	0.00 (0.06)	
	12 Month	0.25 (0.07)	0.07 (0.07)	
Secondary Outcomes				
HVLT-R, delayed recall	Baseline	7.41 (0.36)	6.88 (0.37)	*0.41 (0.02, 0.79)*
	6 Month	7.86 (0.37)	7.04 (0.37)	
	12 Month	7.75 (0.36)	7.77 (0.35)	
HVLT-R, immediate recall	Baseline	23.2 (0.53)	21.86 (0.55)	*0.19 (-0.46, 0.85)*
	6 Month	24.23 (0.6)	22.71 (0.58)	
	12 Month	24.42 (0.63)	23.39 (0.62)	
HVLT-R, recognition	Baseline	11.29 (0.18)	10.91 (0.18)	*0.23 (-0.02, 0.49)*
	6 Month	11.09 (0.19)	10.97 (0.19)	
	12 Month	11.18 (0.16)	11.28 (0.15)	
HVLT-R, retention	Baseline	78.73 (3.48)	74.08 (3.55)	*5.13 (0.73, 9.52)*
	6 Month	80.21 (3.62)	74.69 (3.47)	
	12 Month	79.47 (3.39)	82.71 (3.26)	
Boston Naming Test	Baseline	13.85 (0.19)	13.28 (0.19)	*0.07 (-0.11, 0.25)*
	6 Month	13.65 (0.21)	13.18 (0.21)	
	12 Month	13.84 (0.18)	13.42 (0.18)	

© 2020 Hajjar I et al. *JAMA Network Open.*

Outcome	Visit	Lisinopril (n=89) LS Mean (SE)[a]	Candesartan (n=87) LS Mean (SE)[a]	*Treatment Effect (95% CI)[b]*
Digital Span Test (Backward)	Baseline	5.34 (0.22)	5.13 (0.23)	*0.10 (-0.20, 0.40)*
	6 Month	5.46 (0.26)	5.27 (0.26)	
	12 Month	5.16 (0.26)	5.14 (0.25)	
Digital Span Test (Forward)	Baseline	8.94 (0.24)	9.01 (0.25)	*-0.01 (-0.51, 0.49)*
	6 Month	9.14 (0.26)	9.04 (0.25)	
	12 Month	8.93 (0.66)	9.67 (0.61)	
Dysexecutive Questionnaire	Baseline	13.11 (1.22)	14.97 (1.25)	*-0.72 (-2.20, 0.77)*
	12 Month	12.06 (1.37)	12.49 (1.34)	
Instrumental Activities of Daily Living	Baseline	7.82 (0.18)	7.99 (0.19)	*0.01 (-0.12, 0.14)*
	12 Month	7.82 (0.19)	8.01 (0.19)	
MRI Measures (N=104)				
Total hippocampal volume, mm^3	Baseline	7080.90 (155.74)	7243.95 (162.48)	*3.13 (-57.48, 63.74)*
	12 Month	6955.03 (158.02)	7124.35 (164.30)	
Whole brain cerebral blood flow, ml/100g/min	Baseline	49.03 (1.47)	47.47 (1.36)	*-0.30 (-1.88,1.27)*
	12 Month	47.65 (1.08)	45.48 (1.47)	
White Matter Lesion volume, mm^3	Baseline	4.88 (1.08)	2.45 (1.09)	*-0.31, (-0.64, 0.02)*
	12 Month	5.73 (1.22)	2.68 (1.23)	

Abbreviations: EXAMINER: Executive Abilities: Methods and Instruments for Neurobehavioral Evaluation and Research; HVLT-R: Hopkins Verbal Learning Test - Revised; LS Mean: Least Square Means; SE: standard error; TMT: Trail Making Test.
a: Least square means (standard error) and effect sizes are adjusted for systolic blood pressure and the stratification variables (number of antihypertensive medication and race). TMT B and B-A values are also adjusted for baseline TMT, and hippocampal volumes are also adjusted for intracranial volumes.
b: Treatment effect was derived from MMRM as the solution for the difference in the change (slope) between the candesartan vs. lisinopril arm over the 3 visits considered as a continuous measure (1=baseline,2=6 months, and 3=12 months measurements)

© 2020 Hajjar I et al. *JAMA Network Open.*

eFigure. Serum Potassium and Creatinine Levels Stratified by Treatment Group

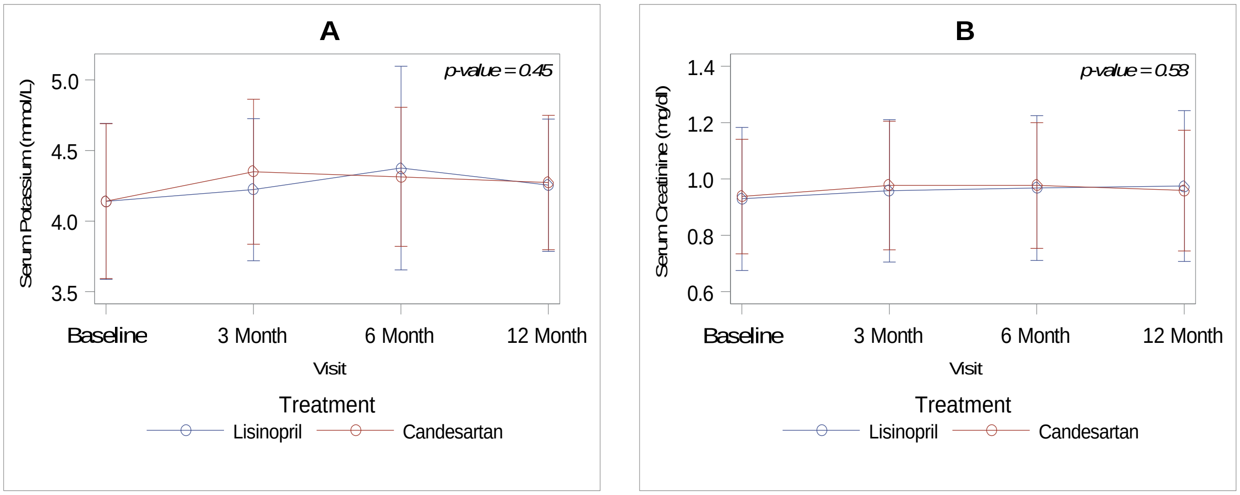

Serum potassium and creatinine measurements and standard deviation (SD) at baseline, 3 months, 6 months and 12 months are illustrated by treatment intervention group. P-value is for the treatment effect lisinopril vs. candesartan.

© 2020 Hajjar I et al. *JAMA Network Open.*
