# Supplementary material for: Effects of Candesartan vs Lisinopril on Neurocognitive Function in Older Adults With Executive Mild Cognitive Impairment: A Randomized Clinical Trial
Source: JAMA Netw Open. 2020 Aug 6;3(8):e2012252. doi: 10.1001/jamanetworkopen.2020.12252 (PMC7411539; doi:10.1001/jamanetworkopen.2020.12252)
Supplement: Supplement 3. — Data Sharing Statement [file jamanetwopen-3-e2012252-s003.pdf]

Data Sharing Statement

Hajjar. Effects of Candesartan vs Lisinopril on Neurocognitive Function in Older Adults With Executive Mild Cognitive Impairment. *JAMA Netw Open*. Published August 06, 2020. 10.1001/jamanetworkopen.2020.12252

Data
Data available: No
